# Characterization of subcutaneous and visceral de-differentiated fat cells

**DOI:** 10.1016/j.molmet.2025.102105

**Published:** 2025-01-28

**Authors:** Yan Li, Houyu Zhang, Carlos F. Ibáñez, Meng Xie

**Affiliations:** 1 Chinese Institute for Brain Research, Zhongguancun Life Science Park, Beijing 102206, China; 2 Academy for Advanced Interdisciplinary Studies, Peking University, Beijing 100871, China; 3 School of Life Sciences, Peking University, Beijing 100871, China; 4 Peking-Tsinghua Center for Life Sciences, Beijing 100871, China; 5 PKU-IDG/McGovern Institute for Brain Research, Beijing 100871, China; 6 Department of Neuroscience, Karolinska Institute, Stockholm 17165, Sweden; 7 School of Psychological and Cognitive Sciences, Peking University, Beijing 100871, China; 8 Beijing Key Laboratory of Behavior and Mental Health, Beijing 100871, China; 9 Biosciences and Nutrition Unit, Department of Medicine Huddinge, Karolinska Institute, Huddinge 14183, Sweden

**Keywords:** Adipocyte de-differentiation, DFAT cell re-differentiation, NF-κB, Diet intervention, scRNA-seq/snRNA-seq, DNA methylation

## Abstract

**Objective:**

The capacity of mature adipocytes to de-differentiate into fibroblast-like cells has been demonstrated in vitro and a few, rather specific in vivo conditions. A detailed comparison between de-differentiated fat (DFAT) cells and adipose stem and progenitor cells (ASPCs) from different adipose depots is yet to be conducted. Moreover, whether de-differentiation of mature adipocytes from classical subcutaneous and visceral depots occurs under physiological conditions remains unknown.

**Methods:**

Here, we used in vitro "ceiling culture", single cell/nucleus RNA sequencing, epigenetic anaysis and genetic lineage tracing to address these unknowns.

**Results:**

We show that in vitro-derived DFAT cells have lower adipogenic potential and distinct cellular composition compared to ASPCs. In addition, DFAT cells derived from adipocytes of inguinal origin have dramatically higher adipogenic potential than DFAT cells of the epididymal origin, due in part to enhanced NF-KB signaling in the former. We also show that high-fat diet (HFD) feeding enhances DFAT cell colony formation and re-differentiation into adipocytes, while switching from HFD to chow diet (CD) only reverses their re-differentiation. Moreover, HFD deposits epigenetic changes in DFAT cells and ASPCs that are not reversed after returning to CD. Finally, combining genetic lineage tracing and single cell/nucleus RNA sequencing, we demonstrate the existence of DFAT cells in inguinal and epididymal adipose depots in vivo, with transcriptomes resembling late-stage ASPCs.

**Conclusions:**

These data uncover the cell type- and depot-specific properties of DFAT cells, as well as their plasticity in response to dietary intervention. This knowledge may shed light on their role in life style change-induced weight loss and regain.

## Highlights

• *In vitro*-derived DFAT cells show lower adipogenic potential and distinct transcriptional profiles compared to ASPCs.
• Inguinal DFAT cells show higher adipogenic potential than those of epididymal origin, which requires NF-κB signaling.
• HFD enhances DFAT cell colony formation and re-differentiation into adipocytes, switching to CD only reverses the latter.
• HFD feeding induces lasting epigenetic changes in DFAT cells and ASPCs that are not reversed by switching to CD.
• DFAT cells are present in inguinal and epididymal adipose depots *in vivo* and resemble late-stage ASPCs.

## 1 Introduction

White adipose tissue (WAT) serves as a major storage site for excess energy in the form of triglyceride and acts as an endocrine organ that secretes various hormones and factors to maintain energy homeostasis. In addition to the white adipocytes that contain triglycerides, WAT also comprises various other cell types in the stromal vascular fraction (SVF), including adipose stem and progenitor cells (ASPCs), immune cells, vascular endothelial cells and small proportions of other cell types [[1], [2], [3]]. Obesity develops when calorie intake regularly exceeds the body's needs, during which WAT expands in an unhealthy way characterized by excessive hypertrophy of existing adipocytes, impaired ASPC differentiation and accumulation of proinflammatory macrophages [4,5]. The overloaded adipocytes are under mechanical stress, become hypoxic and eventually undergo apoptosis, leading to influx of macrophages into the tissue that activates inflammation and efflux of lipids into the neighboring non-adipose organs that causes local and systemic insulin resistance [6]. On the other hand, weight loss via calorie restriction promotes adipocyte size reduction by activating lipolysis and increasing tissue sensitivity to neural and hormonal stimulation [7].

Mature adipocytes have long been considered as a terminally differentiated cell type. Development of the “ceiling culture” method by Sugihara et al. in the late 1980s demonstrated the ability of mature adipocytes to de-differentiate into fibroblast-like cells *in vitro* [8,9]. During de-differentiation, adipocytes undergo morphological changes from round-shaped cells loaded with lipids to spindle-shaped fibroblast-like cells without apparent lipid droplets, accompanied by increased expression of preadipocyte marker genes [10]. The resulting de-differentiated fat (DFAT) cells possess stem cell-like properties and can give rise to multiple cell lineages, including cardiomyocyte, osteoblast and chondrocyte [11,12]. During pregnancy and lactation, adipocytes in mammary gland have been shown to de-differentiate into preadipocyte- and fibroblast-like cells, and subsequently proliferate and re-differentiate into adipocytes after weaning [13]. In addition, reversible de-differentiation of dermal adipocytes is required for hair cycling and skin repair [14,15]. Moreover, adipocyte de-differentiation has been shown to contribute to liposarcoma [16] and mammary tumor progression [17]. So far, the extent to which DFAT cells resemble ASPCs at the functional and transcriptional levels, as well as their plasticity in response to dietary interventions remain to be elucidated.

The family of NF-κB transcription factors has been shown to play important roles in adipose tissue homeostasis and plasticity. This group of proteins encompasses transcriptional activators, such as RelA (p65), RelB and c-Rel, as well as repressors, including NF-κB1 (p50) and NF-κB2 (p52) [18]. Inactive heterodimers of RelA and NF-κB1 are sequestered in the cytosol by IκBα [19]; upon stimulation by cytokines and growth factors, IKKβ kinase becomes activated and phosphorylates IκBα, resulting in the release and nuclear translocation of RelA and NF-κB1. Adipocyte-specific deletion of IKKβ has been shown to exacerbate tissue inflammation induced by HFD [20] and adipocyte cell death, as well as reduce adaptive adipose tissue remodeling and elevate lipolysis in visceral adipose depots [21]. Protein levels of several NF-κB subunits, including RelA, RelB and NF-κB2 have been shown to increase during adipogenesis of 3T3-L1 cells [22].

In the present study, we used cell culture models, single cell/nucleus RNA-sequencing (sc/snRNA-seq) methods, epigenetic analysis and genetic lineage tracing to characterize DFAT cells derived from subcutaneous and visceral adipose depots under different dietary interventions and uncovered the role of NF-κB signaling in regulating the re-differentiation of DFAT cells from inguinal WAT (iWAT).

## 2 Results

### 2.1 DFAT cells derived from subcutaneous and visceral fat depots have lower adipogenic potential and distinct transcriptional profiles compared to ASPCs

Using a previously described *in vitro* “ceiling culture” method [8,9], we obtained DFAT cells from adipocytes isolated from iWAT and epididymal WAT (eWAT) of chow diet (CD)-fed C57BL/6J male mice. Appearance of DFAT cells was characterized by the morphological transition from round to spindle shape and gradual loss of Oil Red O staining (Figure S1A), as well as elevated expression of ASPC marker genes (*Pdgfra* and *Pdgfrb*) and reduced expression of adipocyte marker genes (*AdipoQ* and *Lep*) (Figure S1B). In addition, we crossed the *AdipoQ*^*CreERT2*^ mouse strain that expresses a tamoxifen-inducible Cre recombinase under the control of the *AdipoQ* gene [23] with the Ai14 strain that expresses tdTomato under the control of the *Rosa26* locus in a Cre-dependent manner [24] for genetic labelling of adipocytes. Six-week-old *AdipoQ*^*CreERT2*^;Ai14 mice were injected with three daily consecutive shots of tamoxifen and eWAT adipocytes were collected one day after the last injection for *in vitro* de-differentiation. No overlap between tdTomato and PDGFRA immunostaining that labels ASPCs was observed in whole-mount eWAT tissue sections, confirming the adipocyte-specific labelling of *AdipoQ*^*CreERT2*^ (Figure S1C). tdTomato^+^ adipocytes were found in the isolated adipocyte suspension (Figure S1D), which allows us to specifically follow their *in vitro* de-differentiation process. By monitoring the round-shaped tdTomato^+^ adipocytes in the same visual field on a daily basis, we found that most of the spindle-shaped DFAT cells retained the tdTomato signal (Figure S1E), indicating that these cells are actually derived from the adipocytes. To further exclude a potential contamination of ASPCs, we also derived DFAT cells from adipocytes of *Pdgfra*^*CreERT2*^*;*Ai14 mice whose ASPCs were labelled right before collection by tamoxifen injection. We did not find any tdTomato^+^ cells in such cultures from two independent experiments (data not shown), thereby excluding a potential contamination of ASPCs in the isolated DFAT cells.

**Figure 1:**
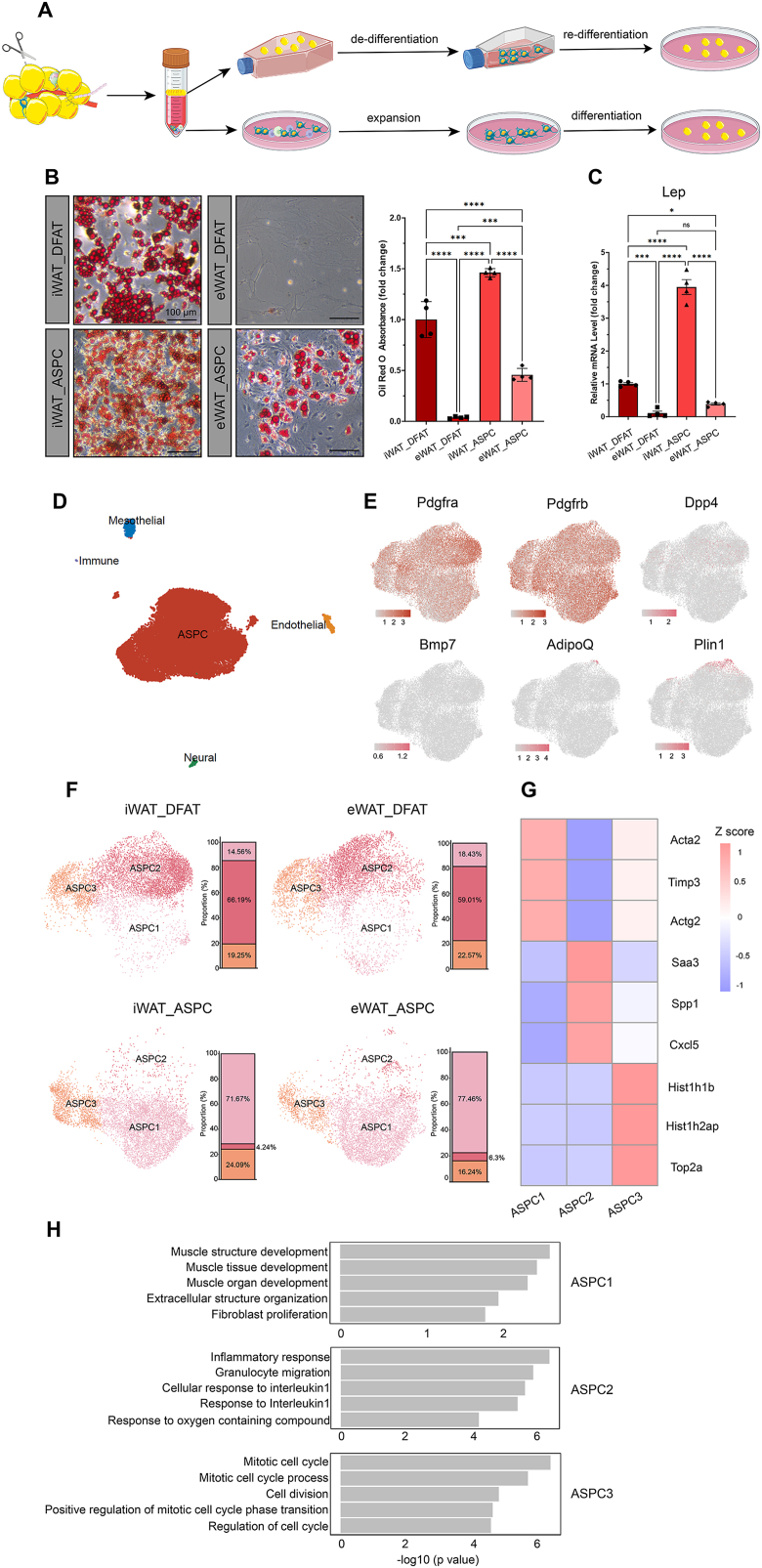
**DFAT cells and ASPCs are different in terms of adipogenic potential and transcriptional profiles**. (A) Schematic illustration of the experimental design for DFAT cell and ASPC *in vitro* culture. (B) Bright field images and quantification of Oil Red O staining of DFAT and SVF cells on DIV 8. n = 4 independent experiments, represented by a dot in the graph. (C) qPCR analysis of *Lep* mRNA level in cells that had been induced for adipocyte differentiation for 6 days. n = 4 independent experiments, represented by a dot in the graphs. Statistical significance was assessed by ordinary one-way ANOVA Tukey's multiple comparisons test in (B–C). ∗*p* < 0.05, ∗∗∗*p* < 0.001, ∗∗∗∗*p* < 0.0001, ns, not significant. Values were normalized to the iWAT_DFAT group and were presented as mean ± standard deviation (SD) in (B–C). (D) Uniform Manifold Approximation and Projection (UMAP) of all cell types in cultured DFAT cells and ASPCs obtained from iWAT and eWAT of C57BL/6J mice fed with CD. (E) Feature plots showing the expression patterns of ASPC and adipocyte marker genes in the ASPC population of the four datasets. Z-score was used for presentation. (F) UMAPs and cellular composition of ASPC subpopulations in the four datasets. (G) Heatmap showing the average expression of DEGs in the three ASPC subpopulations. Z-score was used for presentation. (H) Pathway enrichment analysis of the DEGs of the three ASPC subpopulations using the Gene Ontology-Biological Process gene set. (For interpretation of the references to color in this figure legend, the reader is referred to the Web version of this article).

To compare the adipogenic potential of DFAT cells with ASPCs, we extracted adipocytes and SVF cells in parallel from the same adipose depots (iWAT and eWAT) of CD-fed C57BL/6J mice for de-differentiation and ASPC expansion, respectively, followed by (re)-differentiation into adipocytes (Figure 1A). Six days after induction of *in vitro* differentiation, Oil Red O staining revealed that DFAT cells have a lower adipogenic potential than ASPCs from their cognate fat depot and, among these, cells derived from eWAT always showed lower differentiation potential than those from iWAT (i.e. eWAT_DFAT < eWAT_ASPC < iWAT_DFAT < iWAT_ASPC) (Figure 1B). In agreement with this, *Lep* mRNA levels followed the same trend (Figure 1C). These results suggest that differences in adipogenic potential exist at both the cell type and the depot levels.

To investigate the cellular mechanisms underlying such differences, we applied scRNA-seq to comprehensively compare the transcriptomic profiles of *in vitro*-derived DFAT cells and ASPCs. Following a stringent quality control (Figure S1F), we obtained 7,210, 7,112, 6,481 and 6,686 cells for the iWAT_DFAT, eWAT_DFAT, iWAT_ASPC and eWAT_ASPC groups, respectively. In the integrated datasets, over 90% of the cells were annotated as ASPCs due to their expression of *Pdgfra* (Figure 1D–E). Further clustering revealed three major subpopulations (referred to as ASPC1-3) that were differentially distributed in DFAT cells and ASPCs. Thus, for example, ASPC2 was almost only found in DFAT cells, while ASPC1 was predominantly present in ASPCs (Figure 1F). At the molecular level, ASPC1 specifically expressed genes that are responsible for cytoskeleton and extracellular matrix formation (e.g. *Acta2*, *Timp3* and *Actg2*); ASPC2 was enriched with immune response components (e.g. *Saa3*, *Spp1* and *Cxcl5*) and ASPC3 was characterized by the expression of genes related to epigenetic modification (e.g. *Hist1h1b*, *Hist1h2ap* and *Top2a*) (Figure 1G). Expression of early ASPC (*Dpp4* and *Bmp7*) and adipocyte (*AdipoQ* and *Plin1*) marker genes was barely detected in the analyzed cells, whereas the late-stage ASPC marker gene *Pdgfrb* was expressed in the majority of them (Figure 1E), suggesting that *in vitro*-derived DFAT cells and ASPCs have mostly committed to an adipogenic fate while yet to become mature adipocytes. Pathway analysis on the differentially expressed genes (DEGs) revealed enrichment in muscle development-, immune- and cell cycle-related pathways in the ASPC1, 2 and 3 subpopulations, respectively (Figure 1H).

### 2.2 NF-κB signaling is required for re-differentiation of iWAT DFAT cells

**Figure 2:**
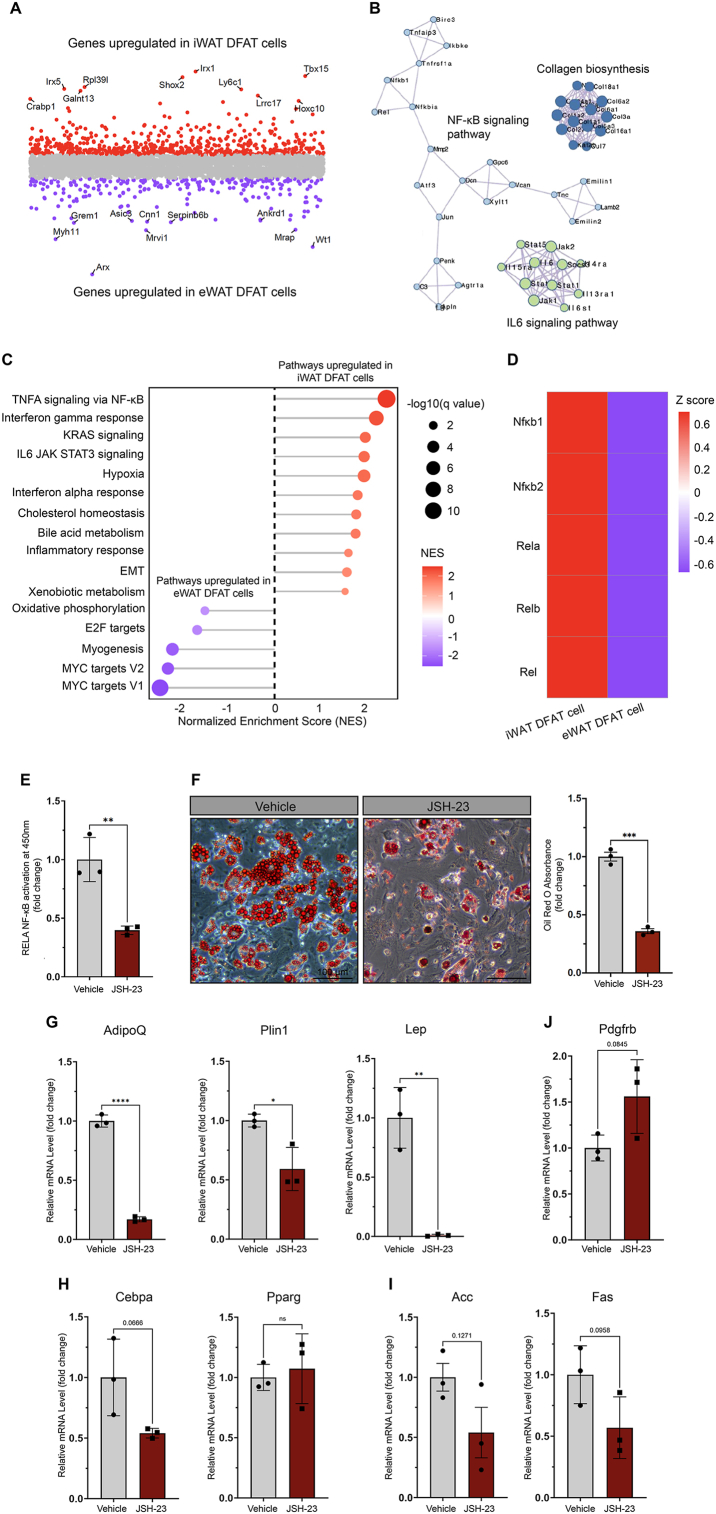
**NF-κB signaling promotes iWAT DFAT cell re-differentiation**. (A) Scatter plot showing the DEGs that were upregulated in iWAT and eWAT DFAT cells. (B) Protein–protein interaction networks functional enrichment analysis of the DEGs that were upregulated in iWAT DFAT cells compared to eWAT. Normalized enrichment score was used for presentation. (C) Hallmark gene set enrichment analysis of the DEGs that were upregulated in iWAT and eWAT DFAT cells. (D) Heatmap showing the expression of the NF-κB component genes in iWAT and eWAT DFAT cells. Z-score was used for presentation. (E) Quantification of active RELA NF-κB in vehicle- and JSH-23-treated iWAT DFAT cells. n = 3 independent experiments, represented by a dot in the graph. JSH-23 was added to the differentiation and maintenance medium at a final concentration of 10 μM. Cells were exposed to JSH-23 at all time during the differentiation and maintenance periods. Details of RELA activation measurement were described in the Materials and Methods section. (F) Oil Red O staining and quantification of re-differentiated iWAT DFAT cells treated by vehicle or JSH-23 on DIV10. n = 3 independent experiments, represented by a dot in the graph. Scale bar, 100 μm. (G–J) qPCR analysis of mRNA levels of mature adipocyte (G), adipogenic (H), lipogenic (I) and ASPC (J) marker genes in vehicle- and JSH-23-treated iWAT DFAT cells. n = 3 independent experiments, represented by a dot in the graph. Statistical significance was assessed by unpaired t-test in (E–J). ∗*p* < 0.05, ∗∗*p* < 0.01, ∗∗∗*p* < 0.001, ∗∗∗∗*p* < 0.0001. *P* values less than 0.2 were also indicated in (H–J). Values of the JSH-23-treated group were normalized to the vehicle-treated group and were presented as mean ± SD. (For interpretation of the references to color in this figure legend, the reader is referred to the Web version of this article).

To explore the molecular mechanisms underlying differences in adipogenic potential between iWAT and eWAT DFAT cells (Figure 1B–C), we identified DEGs that were upregulated in iWAT and eWAT DFAT cells and used those for pathway enrichment analysis (Figure 2A). NF-κB signaling was identified as the top enriched pathway in iWAT DFAT cells compared to eWAT DFAT cells by both protein–protein interaction network functional enrichment analysis (Figure 2B) and gene set enrichment analysis using the Hallmark gene set (Figure 2C). Notably, genes encoding the five members of the NF-κB protein family all had higher expression levels in DFAT cells from iWAT compared to eWAT (Figure 2D). These results prompted us to investigate a possible requirement of NF-κB signaling for iWAT DFAT cell differentiation. To this end, we applied JSH-23, an inhibitor of nuclear translocation of RELA that is critical for NF-κB transcriptional activity [25], to DFAT cells derived from iWAT adipocytes of CD-fed C57BL/6J male mice at the time of induction of differentiation. In parallel with a pronounced reduction in NF-κB activity (Figure 2E), JSH-23 treatment significantly reduced the levels of Oil Red O staining (Figure 2F) and adipocyte marker gene expression in iWAT-derived DFAT cells undergoing adipogenic differentiation (Figure 2G). Likewise, expression levels of key adipogenic (*Cebpa*, but not *Pparg*) and lipogenic (*Acc* and *Fas*) genes were also reduced upon JSH-23 treatment (Figure 2H–I), indicating a potential role for NF-κB signaling in regulating the expression of these genes. Conversely, JSH-23-treated cells showed higher levels of expression of the ASPC marker gene *Pdgfrb*, although statistical significance was not reached (p = 0.0845) (Figure 2J). Together, these results suggest that adipogenic differentiation of iWAT DFAT cells requires NF-κB signaling.

### 2.3 HFD enhances DFAT colony formation capacity and re-differentiation potential, but only the latter is reversed after returning to CD

**Figure 3:**
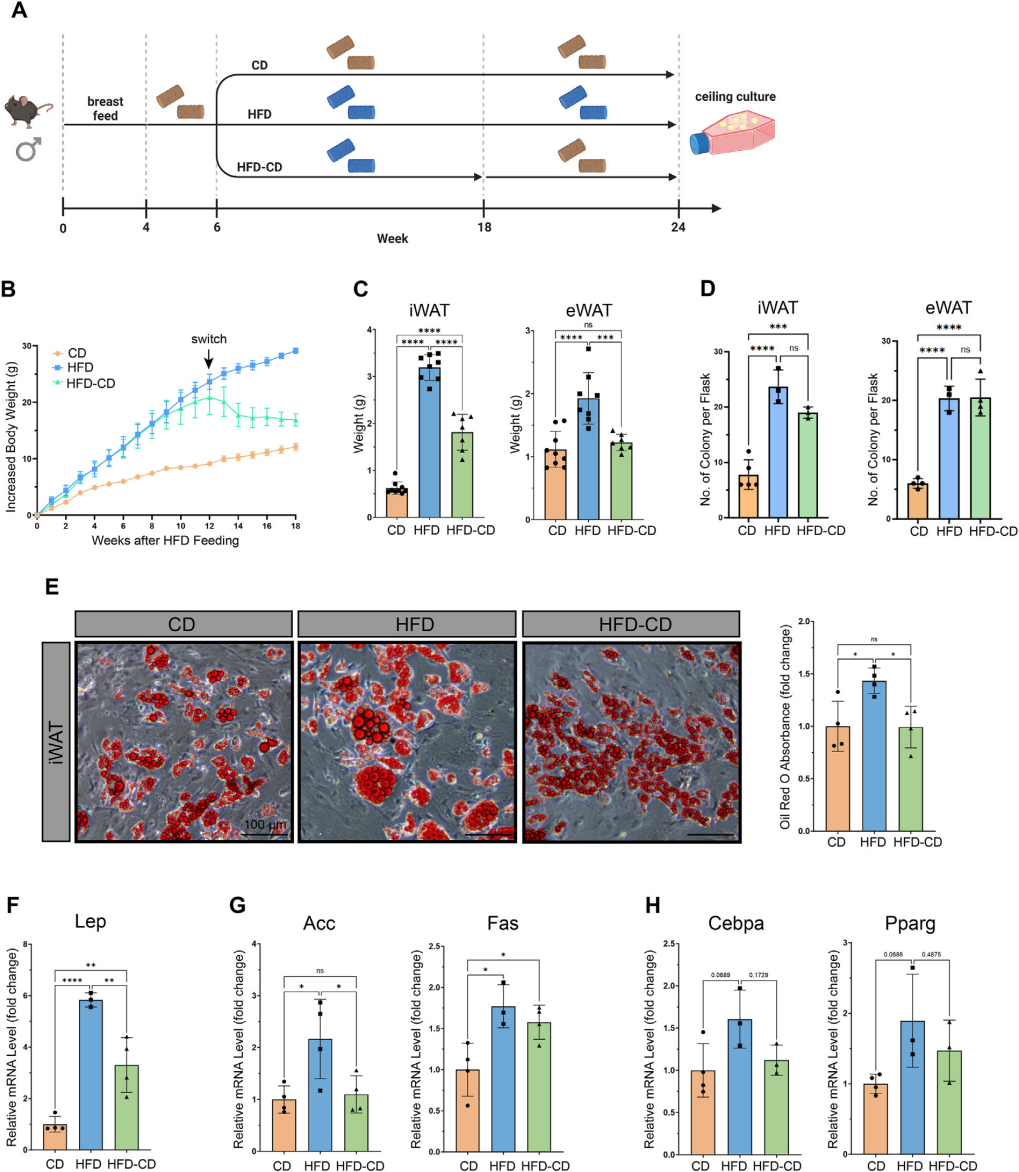
**HFD enhances DFAT cell colony formation and re-differentiation potential, while only the latter is reversed by switching to CD**. (A) Schematic illustration of the three dietary paradigms. Yellow and blue pellets represented CD and HFD, respectively. (B) Body weight change of animals in CD (n = 11), HFD (n = 7) and HFD-CD (n = 7) groups. Values were presented as mean ± standard error of mean (SEM). (C) iWAT and eWAT weights of animals in CD (n = 9), HFD (n = 8) and HFD-CD (n = 7) groups, represented by a dot in the graphs. (D) Comparison of the number of DFAT cell colony among the three diet groups in iWAT and eWAT. n = 3–5 independent experiments, represented by a dot in the graphs. (E) Bright field images and quantification of Oil Red O staining of iWAT DFAT cells from the three diet groups on DIV10. n = 4 independent experiments, represented by a dot in the graph. (F–H) qPCR analysis of mature adipocyte (F), lipogenic (G) and adipogenic (H) marker genes in the DFAT cells of the three diet groups on DIV10. n = 3–4 independent experiments, represented by a dot in the graphs. Statistical significance was assessed by ordinary one-way ANOVA Tukey's multiple comparisons test in (C–H). ∗*p* < 0.05, ∗∗*p* < 0.01, ∗∗∗*p* < 0.001, ∗∗∗∗*p* < 0.0001, ns, not significant. Values were presented as mean ± SD. (For interpretation of the references to color in this figure legend, the reader is referred to the Web version of this article).

Next, we investigated the impact of dietary interventions on adipocyte de-differentiation and subsequent DFAT cell adipogenesis. For this purpose, we placed six-week-old C57BL/6J male mice on CD or HFD feeding for twelve weeks. Subsequently, animals fed with CD were kept on CD for six more weeks (CD group), while animals fed with HFD were either kept on HFD (HFD group) or returned to CD (HFD-CD group) for six more weeks (Figure 3A). As expected, mice that were returned to CD after HFD showed decreased body, iWAT and eWAT weights compared to animals that stayed on HFD (Figure 3B–C). At the end of the feeding period, we collected adipocytes from iWAT and eWAT of the three groups for de-differentiation using the “ceiling culture” method. Although equal numbers of adipocytes were seeded per flask for each dietary condition, significantly less colonies of DFAT cells were observed in the CD group compared to the HFD and HFD-CD groups on days *in vitro* (DIV) 9 (Figure 3D). These results indicate that DFAT cells derived from HFD-fed adipocytes possess higher colony formation potential, which was maintained after reversing back to CD.

Comparing the adipogenic potential of iWAT DFAT cells from the three diet groups, we found that adipocytes derived from DFAT cells of the HFD group had significantly higher lipid content and *Lep* expression than those from the other two (Figure 3E–F). mRNA levels of lipogenic genes (e.g. *Acc* and *Fas*) showed similar trends (Figure 3G). Intriguingly, switching from HFD back to CD reversed the increased expression levels of *Acc*, but not *Fas* (Figure 3G). mRNA levels of adipogenic genes (*Cebpa* and *Pparg*), also increased in the HFD group but just below statistical significance (*p* = 0.0689 and 0.0686, respectively) (Figure 3H). HFD to CD switch reversed this trend, although the change was also not statistically significant (Figure 3H). Together, these results suggest that DFAT cells derived from adipocytes form mice exposed to HFD possess higher re-differentiation potential, while switching to CD after HFD can revert this to the level observed under constant CD.

### 2.4 HFD induces epigenetic changes in DFAT cells and ASPCs that are not reversed after returning to CD

**Figure 4:**
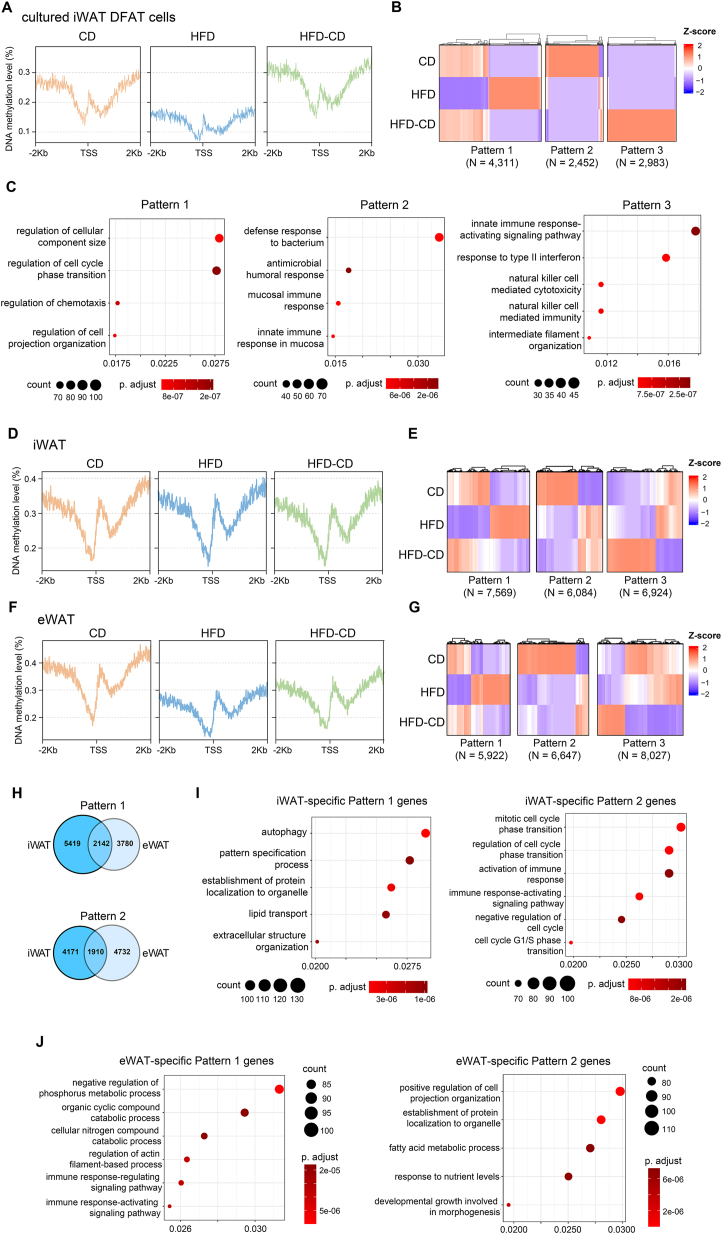
**HFD exposure induces irreversible epigenetic changes in DFAT cells and ASPCs**. (A) Composite plot showing DNA methylation levels around the TSS of 21,936 protein-coding genes in cultured DFAT cells derived from the iWAT adipocytes of the three diet groups. Data were generated from DFAT cells pooled from three independent experiments. A bin size of 10 bp was used to smooth the plot, with averaged methylation levels calculated. TSS, transcription start site. (B) Heatmap showing average DNA methylation levels of 9,746 genes that showed different changing patterns in iWAT DFAT cells among the three diet groups, identified by unsupervised clustering of DNA methylation patterns. Z-score was used for presentation. (C) Bubble charts showing the GO enrichment analysis of Pattern 1 to 3 genes identified in (B). All pathways shown in the chart are among the top 10 enriched pathways in each pattern based on p values. (D, F) Composite plot showing DNA methylation levels around the TSS of 21,936 protein-coding genes in PDGFRA^+^ ASPCs sorted from iWAT (D) and eWAT (F) of the 3 diet groups. Data were generated from ASPCs pooled from 5 animals for all groups. A bin size of 10 bp was used to smooth the plot, with averaged methylation levels calculated. (E, G) Heatmap showing average DNA methylation levels of 20,577 (E) and 20,596 (G) genes that showed different changing patterns in iWAT (E) and eWAT (G) of PDGFRA^+^ ASPCs among the 3 diet groups, identified by unsupervised clustering of DNA methylation patterns. Z-score was used for presentation. (H) Venn diagram showing the overlap between iWAT and eWAT PDGFRA^+^ ASPCs in genes that exhibited changing Pattern 1 and 2. (I–J) Bubble charts showing the GO enrichment analysis of Pattern 1 and 2 genes that were specific to iWAT and eWAT PDGFRA^+^ ASPCs. All pathways shown in the chart are among the top 10 enriched pathways in each pattern based on *p* values.

We investigated whether HFD induces epigenetic changes in DFAT cells compared to CD by performing whole genome bisulfite sequencing (WGBS) on DIV6 DFAT cells derived from iWAT adipocytes of the three diet groups. We observed a dramatic reduction in global DNA methylation (Figure 4A), suggesting more active DNA transcription upon HFD feeding. Notably, overall DNA methylation levels were restored in DFAT cells from the HFD-CD group (Figure 4A). Among the changes observed, three types of patterns were identified in 9,746 genes of the three diet groups: genes that showed either decreased or increased DNA methylation levels upon HFD which were reversed by switching back to CD (Pattern 1, 4,311 genes), genes that showed either decreased or increased DNA methylation levels upon HFD but were not reversed by switching back to CD (Pattern 2, 2,452 genes) and genes that showed increased or decreased DNA methylation levels in the HFD-CD group compared to the other two groups (Pattern 3, 2,983 genes) (Figure 4B). Gene ontology (GO) analysis of the genes in each pattern revealed enrichment of different signaling pathways (Figure 4C). Together, these data suggest that HFD can indeed induce irreversible epigenetic changes in the DFAT cell genome.

To check whether the irreversible HFD-induced epigenetic changes also apply to ASPCs, we collected ASPCs from iWAT and eWAT of the three diet groups by fluorescence-activated cell sorting (FACS) sorting with an antibody against PDGFRA for WGBS analysis. In term of global DNA methylation, iWAT ASPCs did not show obvious changes among the three groups (Figure 4D), while eWAT ASPCs had dramatic reduction upon HFD exposure and was only slightly reversed after switching back to CD (Figure 4F). Similar to the DFAT cells, we also observed three types of DNA methylation changing patterns in the iWAT and eWAT ASPCs (Figure 4E,G). Interestingly, Pattern 1 and 2 genes only showed partial overlaps between the two depots (Figure 4H), suggesting depot-specific epigenetic modifications induced by HFD. GO analysis of Pattern 1 and 2 genes that were specific for iWAT and eWAT revealed enrichment of different pathways (Figure 4I–J). Consistent with iWAT DFAT cells, cellular organization- and immune-related pathways were also highlighted in iWAT Pattern 1 and 2 ASPCs, respectively (Figure 4I). Intriguingly, we found that cellular organization-related pathways were commonly enriched in iWAT Pattern 1 and eWAT Pattern 2 genes, and immune-related pathways were commonly enriched in iWAT Pattern 2 and eWAT Pattern 1 genes (Figure 4I–J), suggesting that the reversible and irreversible epigenetic changes deposited by HFD follow a compensation manner between the two depots. It is worth noting that all pathways shown for the GO analysis of DFAT cells and ASPCs were among the top ten enriched pathways in each category based on their *p* values.

### 2.5 Adipocytes in iWAT and eWAT can de-differentiate into DFAT cells

**Figure 5:**
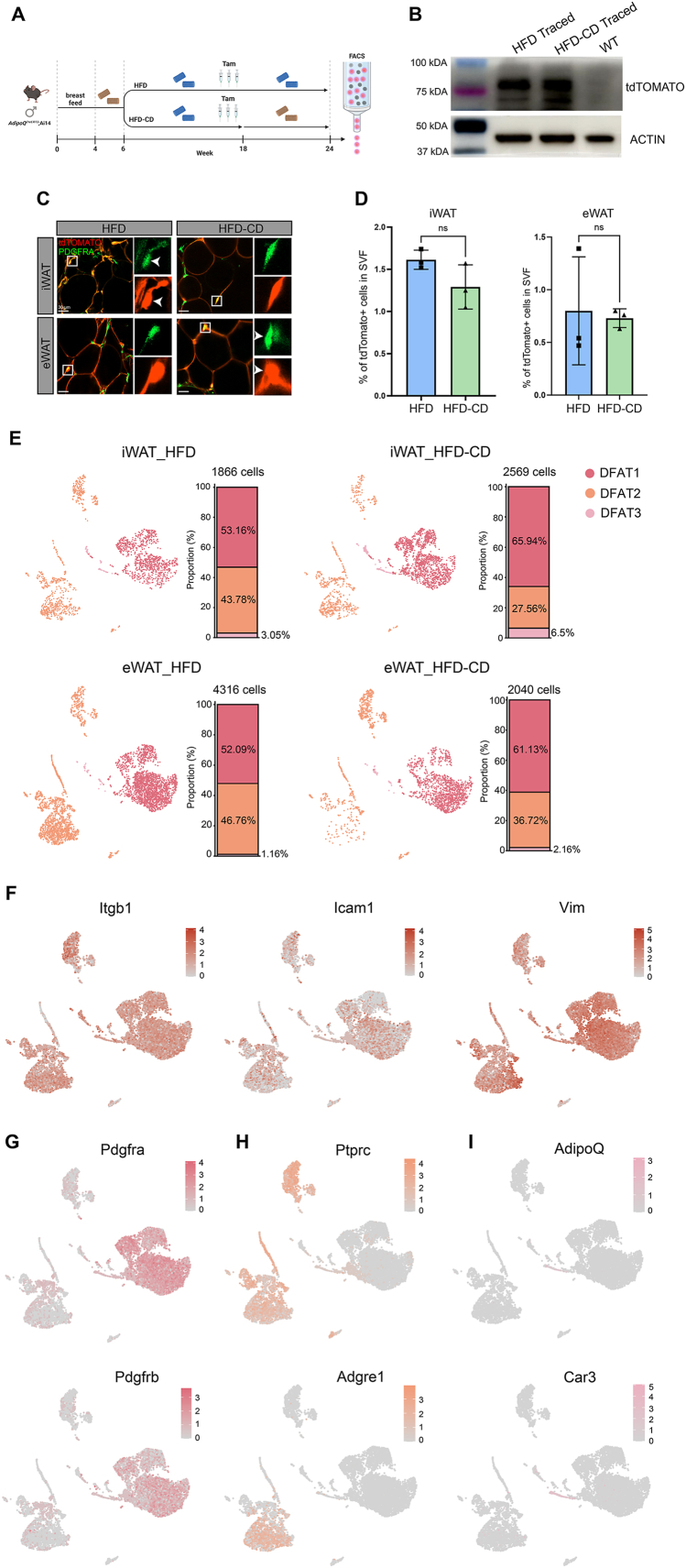
**DFAT cells are present in iWAT and eWAT**. (A) Schematic illustration of the tracing experimental setup in *AdipoQ*^*CreERT2*^;Ai14 male mice. Animals were injected with three consecutive daily shots of tamoxifen at 18-week-old for a subsequent 6-week tracing of the labelled adipocytes. Yellow and blue pellets represented CD and HFD, respectively. (B) Western blot analysis of tdTomato protein level in the SVF of iWAT collected 6 weeks after tamoxifen injection. iWAT SVF of wild type (WT) animals of similar age was used as a negative control. (C) Co-localization of the traced tdTomato signal (red) and the PDGFRA antibody staining (green) in iWAT and eWAT from the two diet groups. Single optical sections were used for illustration to demonstrate the true overlap between the two signals. (D) Percentage of DFAT cells (tdTomato^+^) in SVF of iWAT and eWAT from the two diet groups. n = 3 independent experiments, represented by a dot in the graphs. Statistical significance was assessed by student's t test. ns, not significant. Values were presented as mean ± SD. (E) UMAPs and cellular composition of traced DFAT cell subpopulations in the four datasets. (F–I) Feature plots showing the expression patterns of genes that are commonly expressed in (F) and specific to (G–I) the three DFAT cell sub-clusters. (For interpretation of the references to color in this figure legend, the reader is referred to the Web version of this article).

To assess whether *in vivo* adipocyte de-differentiation occurs in iWAT and eWAT under different dietary intervention, six-week-old *AdipoQ*^*CreERT2*^;Ai14 mice were fed with HFD for twelve weeks before tamoxifen injection and the animals were either kept on HFD (HFD group) or switched to CD (HFD-CD group) for six more weeks to trace the labelled adipocytes (Figure 5A). Four days after injection, labelling specificity in iWAT and eWAT was assessed by collecting SVFs for FACS analysis. Gating parameters for the tdTomato signal were established using SVF cells from C57BL/6J (WT) and *Pdgfra*^*Cre*^*;*Ai14 mice as negative and positive controls, respectively (Figure S2A). In the latter mice, tdTomato is specifically expressed in PDGFRA^+^ ASPCs [26]. Absence of any tdTomato^+^ cells in the SVF indicated that adipocytes, not stem cells or precursors, were specifically labelled (Figure S2B). At the end of the tracing period, SVFs of iWAT and eWAT were collected to assess the expression level of tdTomato. As SVF does not contain mature adipocytes, tdTomato expression detected in this fraction after tracing represents DFAT cells derived from adipocyte de-differentiation. Indeed, tdTomato protein was detected in the SVF at the end of the tracing period (Figure 5B). In agreement with this, overlap between tdTomato and PDGFRA immunostaining was observed in single optical sections of iWAT and eWAT for both dietary groups (Figure 5C), suggesting true co-localization rather than their being in close proximity but different cells. Interestingly, FACS analysis of the SVFs of these animals demonstrated the presence of DFAT cells but without significant differences in their proportion between the HFD and HFD-CD groups for either depot (Figure 5D and S2C-D).

Next, we assessed the transcriptomic profile of the *in vivo*-traced DFAT cells at single cell resolution. Due to the minor fractions of DFAT cells present *in vivo*, we collected the SVFs of iWAT and eWAT from ten to twelve traced animals per group from the HFD and HFD-CD groups for FACS sorting and subsequent scRNA-seq. After filtering (Figure S3A), a total of 10,791 traced DFAT cells were obtained. In the integrated datasets, we revealed three clusters of DFAT cells (DFAT1-3) that expressed progenitor and mesenchymal cell markers, such as *Itgb1*, *Icam1* and *Vim*. (Figure 5E–F). The three DFAT clusters were specifically enriched with typical ASPC (*Pdgfra* and *Pdgfrb*), immune cell (*Ptprc* and *Adgre1*) and adipocyte (*AdipoQ* and *Car3*) markers, respectively (Figure 5G–I). Importantly, expression of *AdipoQ* was absent from the vast majority of DFAT cells (Figure 5I), indicating that these cells are not *AdipoQ*-expressing late-stage preadipocytes but indeed derived from mature adipocytes. (In the former case, FACS sorted cells would have still retained *AdipoQ* expression.) DFAT1 and 2 clusters were the major constituents of the traced DFAT cells, together encompassing over 95% of the population (Figure 5E). Proportion of the immune-like DFAT2 cluster was reduced in the HFD-CD groups of both depots (Figure 5E), which is in line with reduced tissue inflammation after HFD withdrawal. No obvious difference was observed at the depot level (Figure 5E). We also sequenced the traced DFAT cells using the Smart-seq3 method [27] and revealed similar DFAT cell clusters (Figs. S3B–D). Taken together, these results demonstrate that adipocytes in typical subcutaneous and visceral adipose tissues can de-differentiate into DFAT cells expressing ASPC markers *in vivo*.

### 2.6 DFAT cells resemble late-stage ASPCs

**Figure 6:**
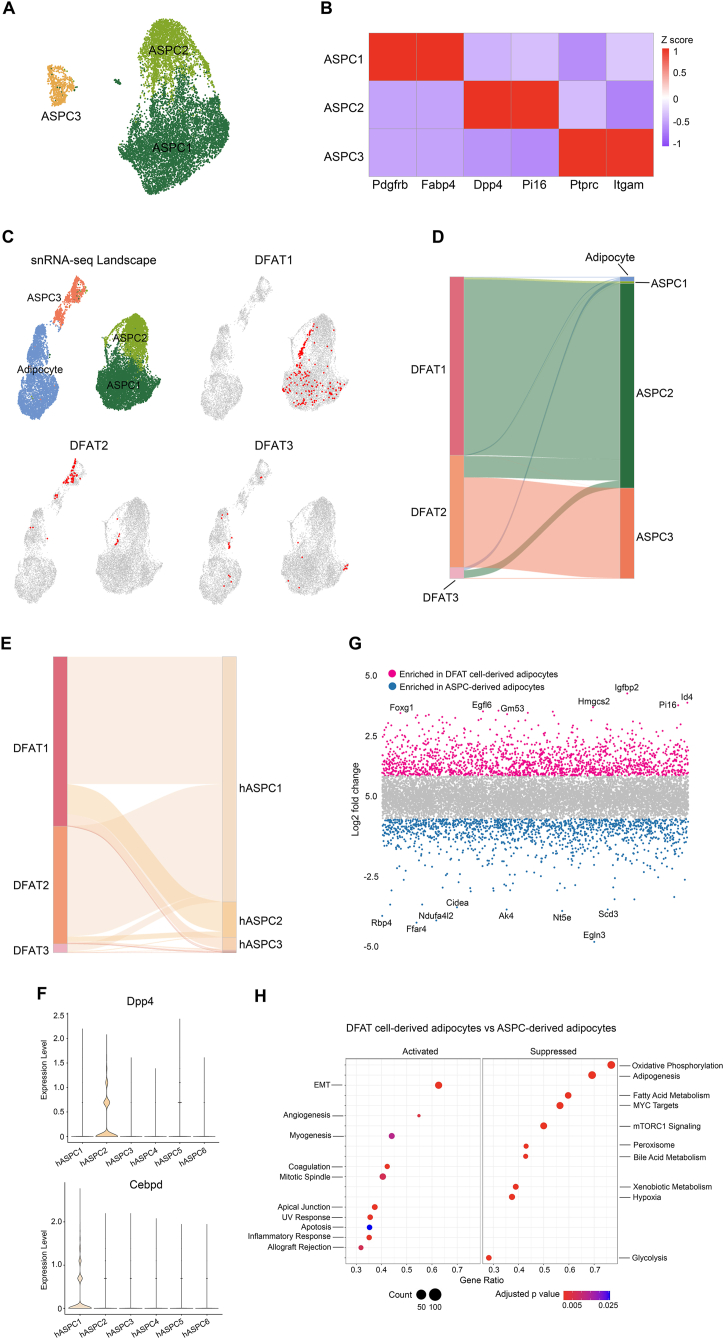
**DFAT cells are similar to late-stage ASPCs**. (A) UMAP showing the ASPC subpopulations in the integrated iWAT and eWAT datasets of HFD and HFD-CD groups. (B) Heatmap showing the DEGs of each ASPC subpopulation. (C) Mapping of DFAT cells from each subpopulation to the whole tissue adipocyte and ASPC landscape. 10% of the DFAT cells were randomly selected from each subpopulation for mapping. (D) Sankey diagram illustrating the similarities between DFAT cell and adipocyte and ASPC subpopulations. (E) Sankey diagram illustrating the similarities between DFAT cell and ASPC subpopulations from human datasets published by Emont et al., 2022 [28]. (F) Violin plots showing the expression levels of *DPP4* and *CEBPD* in the six human ASPC subpopulations identified by Emont et al., 2022 [28]. (G) Volcano plot showing the DEGs that were upregulated in DFAT cell- and ASPC-derived adipocytes. (H) Hallmark gene set enrichment analysis showing the pathways that were up- and down-regulated in DFAT cell-derived adipocytes compared to ASPC-derived adipocytes. EMT, epithelial mesenchymal transition.

To gain a global view on how the DFAT cells are distributed at the whole-tissue level, we performed snRNA-seq on iWAT and eWAT of the HFD and HFD-CD groups and subsequently mapped the traced DFAT cells to the adipocyte and ASPC landscape generated by whole-tissue snRNA-seq. We obtained over 39,000 nuclei in the integrated datasets of iWAT and eWAT of the two diets after filtering (Figure S4A). As expected, proportion of the macrophage population was decreased in both depots after switching from HFD to CD (Figs. S4B–C), in line with reduced tissue inflammation. Notably, eWAT contained much higher fractions of macrophages than iWAT, mainly at the expense of adipocytes (Figure S4C). Further clustering of the ASPC population revealed three sub-populations that expressed late (*Pdgfrb* and *Fabp4*) (ASPC1), early (*Dpp4* and *Pi16*) (ASPC2) and immune cell markers (*Ptprc* and *Itgam*) (ASPC3), respectively (Figure 6A–B). Consistent with the reduced tissue inflammation, the proportion of ASPC3 cells was decreased in the HFD-CD groups of both depots (Figure S4D). Re-clustering of the adipocyte population revealed three subpopulations (Ad1–3) (Figs. S4E–F). Here, the proportion of *Cyp2e1*-expressing Ad2 subpopulation was greatly increased in both depots after switching from HFD to CD (Figure S4G). At the depot level, Ad3 cells expressing the immune cell markers *Ptprc* and *Itgam* comprised much greater proportion of the adipocyte population in eWAT than iWAT (Figure S4G).

From the whole tissue snRNA-Seq dataset, only that corresponding to adipocytes and ASPCs (herein called adipocyte/ASPC dataset) was used for the mapping of DFAT cells. We randomly chose 10% of DFAT cells from each subpopulation to individually look for the cells in the adipocyte/ASPC dataset that had the highest similarity to them. The majority of DFAT1 cells enriched in the expression of typical ASPC markers were mapped to the late-stage ASPC1 subpopulation and the DFAT2 cells mainly fell into the immune marker-expressing ASPC3 subpopulation (Figure 6C–D). The adipocyte-like DFAT3 cells appeared to scatter among the adipocyte and ASPC1 populations (Figure 6C–D). We also mapped our DFAT cells (mouse) to the ASPCs of the integrated human snRNA-seq datasets of subcutaneous and visceral adipose tissues generated by Emont et al., 2022 [28] and found that mouse DFAT cells showed the highest similarity to the late-stage human ASPC1 (hASPC1) subpopulation that was specifically marked by *CEBPD*, but not *DPP4* (Figure 6E–F). These results suggest that DFAT cells traced *in vivo* show greater similarity to progenitors that are at relatively later stages of adipogenesis.

Lastly, we placed the FACS-sorted DFAT cells from iWAT and eWAT of the two diet groups in culture to assess their adipogenic capacity and found that iWAT DFAT cells from the HFD group could efficiently differentiate into adipocytes (Figure S4H). Comparison of the transcriptomic profiles of these iWAT DFAT cell-derived adipocytes with adipocytes derived from ASPCs of the same depot and dietary condition by bulk RNA sequencing revealed differential enrichment in distinct genes and signaling pathways (Figure 6G–H), indicating that these two types of precursor cells can give rise to adipocytes with different properties.

## 3 Discussion

Adipocytes can de-differentiate into ASPC-like cells with multilineage differentiation potential under *in vitro* culture conditions [11,12]. The *in vitro*-derived DFAT cells that we isolated contained distinct ASPC subpopulations compared to cultured ASPCs derived from SVF cells. Intriguingly, markers of early-stage ASPC such as *Dpp4* and *Bmp7* that were identified by a previous study on ASPCs from freshly isolated SVF cells [29] were not expressed in cultured ASPCs or DFAT cells, underscoring the influence of the *in vivo* environment on cellular transcriptomes. A previous study found that ceiling culture-derived DFAT cells from human adipose tissue specimens displayed higher adipogenic potential as well as increased *LEP* and *C/EBPδ* mRNA levels compared to ASPCs from the same source [30]. We also found that adipocyte marker genes were only expressed in a small fraction of the DFAT cells, but not ASPCs. However, mouse ASPCs of either iWAT or eWAT origin had higher adipogenic potential than the respective DFAT cells. Several studies have implicated DFAT cells in the regulation of adipose tissue inflammation [[31], [32], [33]], which could be explained by our observation of a unique subpopulation that expressed several markers related to cells of immune origin. It is also possible that this may account for their lower differentiation potential compared to SVF cells, even though they are at a more advanced stage along the adipogenic process. Our observation that HFD enhances DFAT cell colony formation and re-differentiation is in line with previous findings showing that adipocyte de-differentiation is tightly related to tissue remodeling [[13], [14], [15]]. Interestingly, switching from HFD back to CD reversed the elevated capacity of DFAT cells to re-differentiate, but not DFAT colony formation, suggesting that HFD exposure induces epigenetic changes that cannot be eliminated by diet interventions. Indeed, HFD-induced changes in DNA methylation levels of 10% of the DFAT cell genome were not reversed by switching from HFD to CD. Although *in vitro* de-differentiation may affect DNA methylation, DFAT cells are likely to inherit a fraction of epigenetic marks from the adipocytes that they are derived from.

*In vivo* adipocyte de-differentiation has been demonstrated in mammary gland and dermal adipose tissues that possess high plasticity and undergo extensive remodeling during reproduction and wound healing [[13], [14], [15]]. Here, we identified the presence of DFAT cells in subcutaneous and visceral adipose depots of C57BL/6J mice. Although the DFAT cells that we traced *in vivo* only represented a relatively small fraction of the ASPCs, other stimuli such as repeated alternating cycles of CD and HFD or diets enriched in other components (e.g. protein, carbohydrates) may further affect their abundance. Wang et al., 2018 reported that the transcriptomes of DFAT cells from mammary gland during pregnancy were highly similar to that of endogenous preadipocytes, i.e. with low expression of canonical adipocyte markers (*AdipoQ*, *Fabp4* and *Pparg*) and high expression of preadipocyte and fibroblast markers (*Pdgfrα*, *Pdgfrβ* and *Fap*) [13]. In contrast, Zhang et al., 2019 found that DFAT cells derived during hair follicle recycling were more transcriptomically similar to ASPCs, but with higher adipogenic potential [15]. By tracing iWAT and eWAT adipocytes under HFD feeding and HFD-to-CD switch conditions, we show that the DFAT cells in these depots resembled more closely late-stage ASPCs, which is in line with the properties of the DFAT cells identified by those previous studies.

SVF cells from visceral adipose tissue are known to possess less differentiation potential than those from subcutaneous depots. Such discrepancy has been explained by differences in extracellular matrix composition [34] and ASPC abundance [35]. A previous scRNA-seq analysis revealed a higher proportion of early stage multipotent ASPCs in iWAT compared to eWAT [36]. It has also been shown that SVF cells from mouse eWAT demonstrated poor adipogenic capacity in regular induction medium, which could be improved by addition of bone morphogenetic proteins [37]. In our study, we improved eWAT SVF differentiation by simply reducing the centrifugation speed after collagenase digestion without adding any supplements, suggesting that mechanical force impacts SVF cell isolation. In line with this notion, our scRNA-seq analysis of freshly isolated SVF showed that iWAT and eWAT contain comparable proportions of ASPCs. We attribute this to our gentler SVF isolation protocol compared to other studies, which has allowed us to achieve a greater rate of differentiation from eWAT SVF.

The protein levels of several NF-κB subunits, including RelA, RelB and NF-κB2, as well as NF-κB basal activity have been shown to be upregulated during adipogenesis in 3T3-L1 cells [22]. This is in line with our observation that NF-κB signaling promotes iWAT DFAT cell re-differentiation into adipocytes via its effect on the expression of adipogenic and lipogenic genes. Recently, a SREBP1-mediated lipogenic signal has been shown to be required for canonical NF-κB activation [38], suggesting the existence of reciprocal interactions between the two pathways. The effect on the expression of adipogenic and lipogenic genes might be via a direct binding of NF-κB to their promoter and/or enhancer regions, however, detailed molecular studies are needed to delineate the precise underlying mechanisms. One limitation of the present study is that only pharmacological inhibition of the NF-κB activity was used to assess the function of the pathway in DFAT cell re-differentiation. Manipulations using genetic approaches are needed for further confirmation.

Some drawbacks of the use of tamoxifen-driven CreERT2 in adipocyte lineage tracing studies, including enhanced adipogenesis and prolonged presence of Cre recombinase in the nucleus, have been previously reported [39]. However, we note that the *AdipoQ*^*CreERT2*^ strain used in the present study has been carefully characterized in multiple studies as a valuable model to target mature adipocytes for tracing and gene deletion purposes [23,40], and has been widely used in the area of adipose tissue research [[41], [42], [43], [44], [45], [46]]. Since the focus of our tracing studies is on the adipocyte de-differentiation process, we expect little impact, if any, of any tamoxifen-induced adipogenesis on the experimental outcome. Prolonged nucleus retention of the Cre recombinase could lead to more adipocytes being labeled in an extended period following tamoxifen injection. However, the de-differentiation process is less likely to be altered by these additionally labelled adipocytes, since we focused on the presence of tdTomato^+^ cells in the SVF, not the adipocytes themselves. In addition, a recent study followed the fate of the labelled adipocytes isolated from a tamoxifen-inducible *AdipoQ*^*CreERT*^; mT/mG mouse model *in vitro* and showed that DFAT cells do originate from mature adipocytes [47].

Lifestyle modification circumvents surgery risks and drug safety issues, and is therefore the preferred first-line intervention to combat obesity over bariatric surgery and pharmacotherapy. Together with exercise, dietary intervention is one of the most important strategies of life style modification therapies. Although successful weight loss is commonly observed in obese patients during a period of low-calorie diet, long-term body weight maintenance is more difficult and a weight rebound to sometimes even higher levels often occurs once the dietary intervention is terminated. Human adipocytes have recently been shown to retain epigenetic memories of obesity, contributing to the ‘yo–yo’ effect that is common in weight-loss programs [48]. Our observation of epigenetic changes induced by HFD in both DFAT cells and ASPCs that were maintained after reverting to CD indicates that not only mature adipocytes but also their precursors may be affected by dynamic, diet-mediated epigenetic modifications. These insights may provide strategies for the development of more efficient weight-management interventions.

## 4 Materials and Methods

### 4.1 Animals

All mouse experiments were performed according to the protocol approved by the Institutional Animal Care and Use Committee (IACUC) of Peking University (Psych-XieM-2) and the Chinese Institute of Brain Research (CIBR-IACUC-035). Mice were housed under a 12:12 h light/dark cycle with free access to food and water. *AdipoQ*^*CreERT2*^ (JAX:025124) [23], *Pdgfra*^*Cre*^ (JAX:013148), *Pdgfra*^*CreERT2*^ (JAX:018280) and Ai14 (JAX:007914) [24] strains were purchased from Jackson Laboratory. Tamoxifen (Sigma–Aldrich, T5648) dissolved in corn oil (Solarbio, C7030) was intraperitoneally injected at a dose of 2 mg per animal per day for 3 days to label the adipocytes. All animals used in the present study were male. CD was obtained from Jiangsu Xietong Pharmaceutical Bio-engineering Co., Ltd (Jiangsu, China, 1010097). HFD (60% calorie from fat) was obtained from Research Diet (D12492).

### 4.2 Adipocyte isolation and *in vitro* de-differentiation by “ceiling culture”

Adipose tissue was minced into small pieces and digested in a 0.1% (w/v) type I collagenase solution at 37 °C with shaking at 100 rpm for 45 min. An equal volume of basal culture medium containing 10% serum was added to terminate the digestion. The suspension was filtered through a 400-μm cell strainer. Subsequently, the adipose tissue cells were separated by centrifugation at 186*g* for 10 min, with adipocytes floating in the upper layer and the SVF cells pelleting at the bottom. The adipocytes in the upper layer were collected, washed three times with HBSS solution, and seeded into cell culture flasks. Sufficient culture medium was added to ensure the absence of air in the flasks, creating an anoxic environment for the adipocytes. The culture flasks were inverted to allow the adipocytes to adhere to the top surface of the flask, i.e. the ceiling surface by buoyancy, and incubated at 37 °C with 5% CO_2._ The flasks were flipped back to their normal three days later.

### 4.3 SVF cell isolation and primary culture for adipogenic differentiation

Dissected iWAT and eWAT were washed twice with HBSS solution, before being minced and digested with 1 mg/ml collagenase type I (Sigma–Aldrich, C0130) in digestion buffer containing 0.1 M Hepes sodium salt, 0.12 M sodium chloride, 50 mM potassium chloride, 5 mM d-glucose, 1 mM calcium chloride, and 1.5% bovine serum albumin for 45 min with continuous shaking. After digestion, an equal volume of growth medium containing advanced DMEM/F12 (Gibco, 12634028), 10% FBS, 1% Glutamax (Thermo, 35050061), 1% MEM non-essential amino acids (NEAA, Thermo, 11140050)and 1% Penicillin/Streptomycin (P/S) (Gibco 15140122), was added to terminate the digestion process, before filtering through a 100-μm cell strainer (Fisher Scientific). The filtered cell suspension was subsequently centrifuged at 450*g* for 10 min and resuspended in prewarmed red blood cell lysis buffer for incubation at 37 °C for 5 min. An equal volume of growth medium was added to stop the lysis process, and the cell suspension was filtered through a 40-μM cell strainer (Fisher Scientific) and then centrifuged at 450*g* for 10 min. The cell pellet was resuspended in basal medium and seeded into culture dishes. When cells reached approximately 90% confluence, differentiation medium containing advanced DMEM/F12 supplemented with 5% FBS, 1% Glutamax, 1% NEAA, 1% P/S, 1% Insulin-Transferrin-Selenium (Gibco, 41400045), 33 μM d-biotin (Sigma–Aldrich, B4639), 17 μM pantothenate (Sigma–Aldrich, P5155), 0.5 mM 3-isobutyl-1-methylxanthine (Sigma–Aldrich, I5879), 1 μM dexamethasone (Sigma–Aldrich, D4902), 1 μM rosiglitazone (Sigma–Aldrich, R2408) and 2 nM 3,30,5-Triiodo-L-thyro-9 sodium salt (Sigma–Aldrich, T6397) was added. 3–4 days later, the differentiated cells were maintained in advanced DMEM/F12 supplemented with 2% heat-inactivated FBS, 33 μM d-biotin, 17 μM pantothenate, 1 μM dexamethasone, 10 μg/ml insulin, 2 mM Glutamax, 0.1 mM nonessential amino acids, and 100 U/ml P/S until full differentiation. The maintenance medium was changed every 2–3 days until formation of adipocytes in about 8–9 days after induction.

### 4.4 FACS

SVF cells isolated from subcutaneous and visceral adipose tissues of *AdipoQ*^*CreERT2*^;Ai14 mice were resuspended in 2% FBS dissolved in PBS. 7-AAD solution (Thermo Scientific, A1310) was added, and the samples were subjected to cell analysis and sorting using a Beckman Coulter MoFlo XDP flow cytometer. The samples were loaded and run at a set flow rate, while the instrument's threshold and scatter parameters were adjusted to identify individual cells. Forward scatter (FSC) and side scatter (SSC) were used to assess cell size and internal complexity. Doublets were identified and excluded by comparing the height and width parameters of FSC. Dead cells were effectively discriminated by detecting the fluorescence signal of 7-AAD, ensuring that only live cells were selected for subsequent sorting. Samples isolated from C57BL/6J mice and *Pdgfra*^*Cre*^;Ai14 mice were used as negative and positive controls, respectively. Live cells positive for tdTomato were sorted and subjected to further biological validation and experimental analysis.

For isolation of PDGFRA^+^ ASPCs for WGBS analysis, iWAT and eWAT SVF cells were first incubated on ice for 20 min in 200 μL of 2% FBS/PBS containing anti-mouse CD16/CD32 Fc Block (clone 2.4G2, BD) at a 1:200 dilution. Cells were then incubated with CD140a (PDGFRA) Antibody-APC (Thermo Fisher, 17-1401-81) at a 1:200 dilution while rotating at 4 °C for 30 min. Following incubation, cells were washed three times with 2% FBS/PBS and stained with 7-AAD to exclude dead cells.

### 4.5 Immunofluorescence staining

Mice were anesthetized and perfused with PBS prior to adipose tissue dissection. After overnight fixation in 1% paraformaldehyde at 4 °C, the samples were embedded in 5% low-melting point agarose (Solarbio, 9012-36-6) and sectioned into 100 to 400-μm-thick slices with a Vibratome (Leica, VT1200). Tissue slices were permeabilized in PBST supplemented with 5% donkey serum and 0.3% Triton X at room temperature for 30 min. Following antibodies were used: PDGFRA antibody, 1:200 (Cell Signaling, 3174); Donkey anti-rabbit Alexa Fluor 488 IgG H&L, 1:1000 (Invitrogen, A31573). Images were taken with an Airyscan 2 LSM 900 confocal microscope (ZEISS) and analyzed using the Imaris software (Bitplane version 9.0.1).

### 4.6 Bulk RNA sequencing and data analysis

Bulk RNA sequencing was outsourced to Novogene Co., Ltd, Beijing, China. Total RNA quantity and quality were assessed using the Agilent 2100 Bioanalyzer with the RNA Nano 6000 Assay Kit (Agilent Technologies, CA, USA) according to the manufacturer's instructions. Total RNA was used as the starting material for library construction. mRNA with poly(A) tails was enriched using Oligo(dT) magnetic beads, and the obtained mRNA was randomly fragmented in fragmentation buffer containing divalent cations. Using the fragmented mRNA as a template and random oligonucleotides as primers, the first-strand cDNA was synthesized in an Moloney Murine Leukemia Virus (M-MuLV) reverse transcriptase system (New England Biolabs). Subsequently, the RNA strand was degraded by RNaseH (New England Biolabs), and the second-strand cDNA was synthesized using dNTPs as substrates in a DNA polymerase I system (Promega). The purified double-stranded cDNA underwent end repair, A-tailing, and sequencing adapter ligation. cDNA fragments ranging from 370 to 420 bp were selected using the AMPure XP beads (Beckman Coulter), followed by PCR amplification. The PCR products were further purified using the AMPure XP beads to obtain the final library. After library construction, initial quantification was performed using a Qubit 2.0 Fluorometer, and the library was diluted to 1.5 ng/μl. Subsequently, the insert size of the library was assessed using an Agilent 2100 Bioanalyzer. Once the insert size met the expected range, qRT-PCR was employed to accurately quantify the effective library concentration for library quality control with a concentration higher than 2 nM. After quality check, different libraries were pooled according to their effective concentrations and the desired output data volume. The pooled libraries were then sequenced using an Illumina NovaSeq 6000 platform.

The obtained fastq files were initially subjected to quality assessment using fastQC to remove low-quality and adapter sequences with Trimmomatic. The processed reads were then aligned to the mouse reference genome mm10 using the STAR software, generating sorted BAM files. Read counting was performed using the featureCounts function from the Subread package to obtain the expression matrix, which was further analyzed in R. The edgeR package was employed to quantify gene expression levels and calculate normalized counts, applying library size normalization and trimmed mean of M-values (TMM) calculation. Following normalization, differential expression analysis was conducted between SVF and DFAT samples. Log2 fold changes were calculated, and genes were classified as upregulated or downregulated based on log2 fold change thresholds of greater than 1 or less than −1, respectively. Gene expression data were then merged with external gene annotation data to map gene symbols to their corresponding Entrez IDs.

### 4.7 Nuclei isolation for snRNA-seq

Nuclei isolation from adipose tissue was performed based on a published protocol [49]. After dissection, iWAT and eWAT were minced with scissors in 500 ml nuclei isolation buffer (NIB) that contains 250 mM sucrose (Solarbio, S8271), 10 mM HEPES (Solarbio, H8090), 1.5 mM MgCl_2_ (Sigma–Aldrich, M8266), 10 mM KCl (Sigma–Aldrich, P5405), 0.001% NP-40 (Sigma–Aldrich, I3021), 0.2 mM DTT (Sigma–Aldrich, D9779), and 1 U/μl RNase inhibitor (Solarbio, R8061) in DEPC-treated water. The samples were then homogenized with a 2 ml Dounce homogenizer (Sigma–Aldrich, D8938), before filtering through a 70-μm cell strainer (Falcon, 352350). Nuclei pellet was collected by centrifugation at 500*g* for 5 min at 4 °C and was subsequently resuspended in nuclei resuspension buffer that contains 2% BSA (Sigma–Aldrich, A1933), 1.5 mM MgCl_2_, and 1 U/μl RNase inhibitor in PBS. All solutions were sterile filtered prior to use.

### 4.8 Single cell isolation for scRNA-seq

Adherent cells were gently washed 3 times with pre-cooled PBS to remove residual culture medium and debris. 0.25% Trypsin–EDTA (Gibco, 25200-056) was added and incubated at 37 °C for 3–5 min to detach the cells. Cell suspension was collected by centrifuging at 450*g* for 10 min and was subsequently resuspended in sorting buffer supplemented with 2% FBS (Gibco).

### 4.9 snRNA-seq and scRNA-seq library construction and sequencing

snRNA-seq and scRNA-seq library construction and sequencing were outsourced to ©Analytical Biosciences Limited, Beijing, China. Isolated nuclei were sorted on a BD FACSMelody4-Way Cell Sorter (BD Biosciences) with a 100-μm nozzle after staining with 10 μg/ml 7-AAD (Invitrogen, A1310). Isolated cells were stained with 7-AAD at a concentration of 5 μg/ml and subjected to FACS sorting. Number of nuclei and cells were quantified with a Countstar Rigel S5 Automated Cell Counter. Approximately 20,000 nuclei or cells were applied to the Chromium microfluidic chips with 3′ v3 chemistry and barcoded with a 10x Chromium Controller using the Single-Cell 3′ Reagent Kit v3.1 (10x Genomics). The following program was used for GEM-Reverse Transcription: 53 °C for 45 min, 85 °C for 5 min, and held at 4 °C. The resulting emulsions were lysed for cDNA isolation and purification with the Cleanup Mix containing DynaBeads and SPRIselect reagent (Thermo Scientific) and amplified with PCR. cDNA quality was assessed with an Agilent 2100 Bioanalyzer. cDNA amplicon size was optimized by enzymatic fragmentation and size selection. P5, P7, i7, and i5 sample indexes and TruSeq Read 2 (read 2 primer sequence) were added by end repair, A-tailing, adapter ligation, and PCR, respectively. Final libraries containing the P5 and P7 primers were sequenced by an Illumina NovaSeq 6000 sequencer with 150 bp paired-end reads, aiming for a coverage of approximately 50,000 raw reads per nucleus/cell.

### 4.10 snRNA-seq and scRNA-seq data analysis

Raw fastq sequencing data were processed using Cell Ranger 7.0.0 with the “--include-introns” option enabled to include reads mapping to the introns annotated in Ensembl, and the mm10 mouse reference genome was utilized to quantify gene expression levels. The raw nuclear gene count matrices were subsequently processed with CellBender 0.2.2 [50] to eliminate ambient RNA contamination and empty droplets. The purified count matrices were then analyzed using Seurat 4.3.0 [51] for downstream analysis. During data preprocessing, quality control thresholds were applied to filter out low-quality cells, with criteria including a gene detection count ranging from 200 to 7500, UMI counts between 500 and 75,000, and mitochondrial reads comprising less than 10% of total reads. Cells expressing fewer than 10 nuclear genes were also removed. Additionally, DoubletFinder 2.0.3 was employed to exclude nuclei doublets [52].

To correct for inconsistencies in sequencing depth among cells, a series of data preprocessing and normalization steps were implemented. The LogNormalize method was employed for normalization, whereby gene expression counts for each cell were divided by the total expression counts of that cell, multiplied by a scaling factor of 10,000, and subsequently log-transformed. Highly variable genes (HVGs) were identified using the variance stabilizing transformation (VST) method. The ScaleData function was used for scaling and centering the data, where each gene feature was centered and standardized to ensure a mean of zero and a standard deviation of one. To further mitigate the variations arising from technical errors and biological differences, the sctransform method was applied to perform regularization using a negative binomial model [53,54].

To integrate multiple datasets and mitigate batch effects, the Harmony algorithm was utilized to analyze and adjust principal components to align data from different batches, thereby reducing variations introduced by non-biological factors such as experimental conditions and processing times. This method effectively identified and corrected batch effects in multidimensional space while preserving biological variability, facilitating unbiased integration of cells across different datasets [55,56].

DEGs were calculated using the ‘FindMarkers’ function in Seurat. The criteria were established requiring a log2 fold change (log2FC) greater than 0.25, an adjusted P-value less than 0.05, and expression of the gene in more than 25% of cells. Cell types were then annotated based on biological characteristics using references, classic cell-type-specific marker genes, and top marker genes identified from the PanglaoDB database. Similarly, the ‘FindMarkers’ function was employed to identify differentially expressed genes under various experimental conditions. Subsequently, gene set enrichment analysis was conducted using the ‘gsva’ function from the GSVA package, which calculated the enrichment scores for each sample. The resulting enrichment scores, indicative of the relative activity of each gene set within the samples, were visualized using the ‘Pheatmap’ function.

In the cluster similarity analysis, the top 50 DEGs for each cell type were selected from the reference dataset. The average expression values of these genes were then calculated across the corresponding cell types in the target dataset, serving as the basis for determining similarity scores between the cell types of the target and reference datasets. Following the computation of similarity scores between each DFAT cell and the various nuclear subtypes, the subtype with the highest score was identified as the most closely matching cell subtype. Sankey diagram was used to visually represent the label transfer.

### 4.11 Oil Red O staining and quantification

Cell culture medium was removed and the cells were washed three times with PBS. A sufficient amount of 4% formaldehyde solution was added to the cells, ensuring complete coverage of the cell layer, and the cells were fixed for 10 min at room temperature. Then, Oil Red O staining solution (Sigma–Aldrich, O1391) was added to each well in a quantity sufficient to cover the cell layer. The plate was gently shaken at room temperature for 1 h to promote thorough contact between the stain and the cells. The cells were rinsed with distilled water at least three times to remove unbound Oil Red O solution until the wash solution was clear. At this point, the stained cells were imaged and analyzed using an Axio Observer inverted microscope (Zeiss). After removing the remaining distilled water, 100% isopropanol was added to each well and incubated at room temperature for 15 min to elute the bound Oil Red O. The absorbance was measured at a wavelength of 500 nm using a multifunctional microplate reader to quantify the Oil Red O content within the cells.

### 4.12 RNA isolation and qPCR analysis

After dissection, adipose tissues were snap-froze in liquid nitrogen before extracting total RNA with the TRIzol reagent (Invitrogen, 15596018). 1 μg RNA was used for cDNA synthesis using the H Minus cDNA first-strand synthesis kit (Thermo Scientific, K1652). qPCR was performed with the PowerUp SYBR TM Green Master Mix (Applied Biosystems, A25742) using a CFX96TM Real-Time PCR Detection System (Bio-Rad). Cq values were normalized to levels of Ywhaz using the ΔΔ-Ct method. The following mouse gene primers were used:

*Acc*

forward 5′-ATGGGCGGAATGGTCTCTTTC-3′,

reserve 5′-TGGGGACCTTGTCTTCATCAT-3′;

*Fas*

forward 5′-TGCTCCCAGCTGCAGGC-3′,

reserve 5′-GCCCGGTAGCTCTGGGTGTA;

*Cebpa*

forward 5′-CAAGAACAGCAACGAGTACCG-3′,

reserve 5′-GTCACTGGTCAACTCCAGCAC-3′;

*Pparg*

forward 5′-GTACTGTCGGTTTCAGAAGTGCC-3′,

reserve 5′-ATCTCCGCCAACAGCTTCTCCT-3′;

*Lep*

forward 5′-GAGACCCCTGTGTCGGTTC-3′;

reserve 5′- CTGCGTGTGTGAAATGTCATTG-3′;

*AdipoQ*

5′-GAAGCCGCTTATGTGTATCGC-3′;

reserve 5′-GAATGGGTACATTGGGAACAGT-3′;

*Pdgfrb*

forward 5′-AGACACTGGGGAATACTTTTGTG-3′;

reserve 5′-CGGCCCTAGTGAGTTGTTGT-3′;

*Dgat2*

forward 5′- CTGTGCTCTACTTCACCTGGCT-3′,

reserve 5′- CTGGATGGGAAAGTAGTCTCGG-3′;

*Pdgfra*

forward 5′-TCCATGCTAGACTCAGAAGTCA-3′;

reserve 5′-TCCCGGTGGACACAATTTTTC-3′.

### 4.13 WGBS library preparation

WGBS library preparation was outsourced to Novogene Co., Ltd, Beijing, China. Following sample qualification, 100 ng of genomic DNA was mixed with 0.5 ng of unmethylated lambda DNA and subsequently sheared into 200–400 bp fragments using a Covaris S220 ultrasonicator (Covaris, USA). After fragmentation, unmethylated cytosine was converted to uracil using the EZ DNA Methylation-GoldTM Kit (Zymo Research). This was followed by adapter ligation, fragment selection, and PCR amplification to construct the library. Library quality was assessed using the Agilent 5400 system (Agilent, USA) and quantified by QPCR with a required concentration exceeding 1.5 nM. Libraries that passed quality control were sequenced on an Illumina platform (Illumina, CA, USA).

### 4.14 WGBS data analysis

The quality of raw sequencing data was assessed using FastQC. Adapter sequences and low-quality ends in the Illumina sequencing data were subsequently removed with the Trim Galore tool. Processed reads were then aligned to the mm10 mouse reference genome using Bismark (version 0.23.1). Single-base methylation levels were extracted from deduplicated BAM files using the ‘bismark_methylation_extractor' command, and output bedgraph files were converted to bigWig format for downstream analysis. DNA methylation levels within ±2 kb around transcription start sites (TSS) of all 21,936 protein-coding genes were calculated using Deeptools (version 3.5.3) via the ‘computeMatrix' function. Tab files were used for composite plots via the ‘plotProfile' function or custom analysis in R. For genome-wide analysis, average DNA methylation levels within ±1 kb around all 21936 TSS were calculated. DNA methylation patterns were determined using k-means clustering and 2142 genes with lower methylation levels in HFD and diet-switching group were extracted for heatmap representation and GO analysis. Average DNA methylation levels within ±1 kb around TSS of 39 genes enriched in the “NF-кB signaling pathway” were presented as boxplot.

### 4.15 Assessment of NF-κB activity

To assess NF-κB activation, we employed the TransAM® NF-κB Family Transcription Factor Assay Kit (Active Motif, 43296), which utilized an ELISA-based approach to detect and quantify the activation of NF-κB transcription factors. The kit contains a 96-well plate that was pre-coated with oligonucleotides containing the NF-κB consensus binding site. Activated NF-κB homodimers and heterodimers in nuclear or whole-cell extracts specifically bound to these oligonucleotides. Detection of the bound NF-κB subunits p65 was facilitated by antibodies specific to these subunits, followed by a secondary antibody conjugated to horseradish peroxidase. Quantitative measurement was performed by spectrophotometry at 450 nm.

### 4.16 Western blot

Protein was extracted by adding 0.3 ml 100% ethanol to the interphase and lower organic phase of the TRIzol lysate and centrifuged at 2000*g* for 5 min at 4 °C. The upper phase was collected for protein precipitation with isopropanol by centrifuging at 12,000*g* for 10 min at 4 °C. Protein pellet was washed twice with 0.3 M guanidine hydrochloride (Solarbio, G8070) dissolved in 95% ethanol, once in 100% ethanol and air-dried, before being denatured in 200 μl 1% SDS (Solarbio, S8010) at 50 °C. Protein concentration was determined using a BCA protein assay kit (Solarbio, PC0020). 15 μg protein was loaded onto an SDS-PAGE gel and subsequently transferred to a PVDF membrane (Bio-Rad, 1620177). The blots were treated with chemiluminescent HRP substrate (Millipore, WBKLS05000) and imaged using an Amersham ImageQuant 800 protein blot imaging system. Band intensity was quantified using ImageJ version 1.53k. The following antibodies were utilized at the indicated working concentrations: β-Actin at 1:1000 dilution (Abcam, ab8227), RFP at 1:1000 dilution (Abcam, ab62341), HSL at 1:1000 dilution (Cell Signaling, 4107), and phospho-HSL at 1:1000 dilution (Cell Signaling, 45804).

### 4.17 Smart3 sequencing library construction and sequencing

Construction of sequencing libraries was performed according to the Smart-seq3xpress protocol [27]. Briefly, cells were isolated by FACS into 3 μl lysis buffer containing 0.125 μM oligo-dT primer, overlaid with 4 μl Silicon Oil (5 cSt; Sigma–Aldrich, 317667). Reverse transcription was performed in a 4-μl reaction volume using 0.75 μM Smart-seq3 template-switching oligonucleotide (TSO). cDNA preamplification was conducted in 10-μl reactions using SeqAmp polymerase (Takara Bio) for 18 cycles. The amplified cDNA was diluted 1:10 with nuclease-free water, and 1 μl of the diluted product was used for tagmentation with in-house Tn5 enzyme. Indexed libraries were pooled into 96-well plate, purified, and sequenced on an Illumina NovaSeq 6000 platform.

### 4.18 Smart3 sequencing data analysis

The kallisto-bustools workflow was employed to process the Smart-seq3 single-cell RNA sequencing data [57]. Raw sequencing reads were pseudo aligned to a reference transcriptome using kallisto (v0.48.1) with parameters optimized for Smart-seq3 data. Gene-level count matrices were subsequently generated using the bustools (v0.42.0) count command. The resulting count matrices were then prepared for downstream analyses in R. Cells with nFeature between 100 and 6000 and percent.mt less than 10% were retained. The filtered count matrix was processed using the recommended pipeline with default parameters, including the “SCTransform”, “RunPCA”, “RunUMAP”, “FindNeighbors”, and “FindClusters” functions. Multiple datasets were integrated and analyzed using the Harmony package. The UMAP plots in the study were generated using the plotting functions from the Seurat package. The sorted DFAT cell subpopulations were annotated using known marker genes for adipose tissue cell types.

### 4.19 Statistics

Statistical analyses were performed using GraphPad Prism version 8.0.0. Ordinary one-way ANOVA Tukey's multiple comparisons test and unpaired t test were used to test statistical significance. The level of statistical significance was assigned as ∗*p* < 0.05, ∗∗*p* < 0.01, ∗∗∗*p* < 0.001, ∗∗∗∗*p* < 0.0001.

## CRediT authorship contribution statement

**Yan Li:** Methodology, Investigation. **Houyu Zhang:** Methodology, Investigation. **Carlos F. Ibáñez:** Writing – review & editing, Supervision, Funding acquisition, Conceptualization. **Meng Xie:** Writing – review & editing, Writing – original draft, Supervision, Investigation, Funding acquisition, Conceptualization.

## Declaration of competing interest

The authors declare that they have no known competing financial interests or personal relationships that could have appeared to influence the work reported in this paper.

## Data Availability

All data were in GEO database under the accession number of GSE272938. All codes were in https://github.com/YanLi0519/Dedifferentiation_project.

## Appendix A Supplementary data

Multimedia component 1 Figure S1. Characterization of adipocyte de-differentiation *in vitro*. (A) Oil Red O staining of the adipocytes and DFAT cells along the de-differentiation process. Scale bar, 100 μm. (B) qPCR analysis of adipocyte (AdipoQ and Lep) and ASPC (Pdgfra and Pdgfrb) marker genes in iWAT (B) and eWAT (C) DFAT cells. Statistical significance was assessed by ordinary one-way ANOVA. ∗*p* < 0.05, ∗∗*p* < 0.01, ∗∗∗*p* < 0.001, ∗∗∗∗*p* < 0.0001. n = 4 independent flasks from 2 independent experiments, represented by a dot in the graph. Values were normalized to the DIV2 group and were presented as mean ± SD. (C) PDGFRA antibody staining (green) and tdTomato signal (red) in whole-mount eWAT tissue section of AdipoQCreERT2; Ai14 mice. Scale bar, 40 μm. (D) Presence of tdTomato + adipocytes in the upper layer of digested eWAT tissue of AdipoQCreERT2; Ai14 mice. Scale bar, 100 μm. (E) Daily monitoring of tdTomato + adipocytes during the *in vitro* de-differentiation process. The same visual field was followed in all images. DIV4 was one day after flipping the flask. Scale bar, 100 μm. (F) Quality controls of scRNA-seq of *in vitro*-derived DFAT cells and ASPCs. UMI, unique molecular identifier. Multimedia component 1

Multimedia component 2 Figure S2. FACS analysis of the traced DFAT cells (A) FACS gating setup with SVF cells from WT animals that do not express any tdTomato signal and *PdgfraCre*;Ai14 animals where all their ASPCs express tdTomato signal. (B–D) FACS analysis of tdTomato^+^ DFAT cells 4 days (B) or 6 weeks (C–D) after tamoxifen injection. Numbers in the diagrams indicate the percentage of the gated cell population in (A–D). Multimedia component 2

Multimedia component 3 Figure S3. Analysis of traced DFAT cells by Smart-seq3. (A–B) Quality controls of traced DFAT cells analyzed by scRNA-seq (A) and Smart-seq3 (B). (C) UMAP of the three DFAT cell sub-populations analyzed by Smart-seq3. (D) Feature plots showing the Smart-seq3-analyzed expression patterns of the same genes as in Figure 5F–I. Multimedia component 3

Multimedia component 4 Figure S4. Whole-tissue snRNA-seq analysis of iWAT and eWAT. (A) Quality controls of snRNA-seq of iWAT and eWAT from the two diet groups. (B) Heatmap showing the DEGs of each cell population in the integrated whole tissue snRNA-seq datasets from the two diet groups of iWAT and eWAT. (C) UMAPs and composition of each cell population in the four groups. (D) UMAPs and composition of each ASPC sub-population in the four groups. (E) UMAP of adipocyte sub-populations. (F) Heatmap showing the DEGs of each adipocyte sub-population. (G) UMAPs and composition of each adipocyte sub-population in the four groups. (H) Bright field images of DFAT cells sorted from iWAT and eWAT of the two diet conditions that have been cultured for adipocyte differentiation for 8 days. Scale bar, 100 μm. Multimedia component 4
