# Supplementary figures and images for: Characterization of subcutaneous and visceral de-differentiated fat cells
Source: Mol Metab. 2025 Jan 28;93:102105. doi: 10.1016/j.molmet.2025.102105 (PMC11848481; doi:10.1016/j.molmet.2025.102105)

A

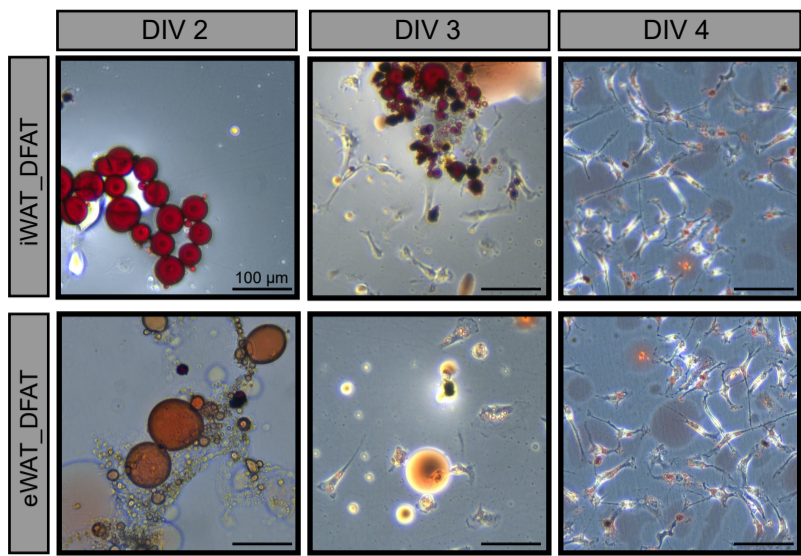

B

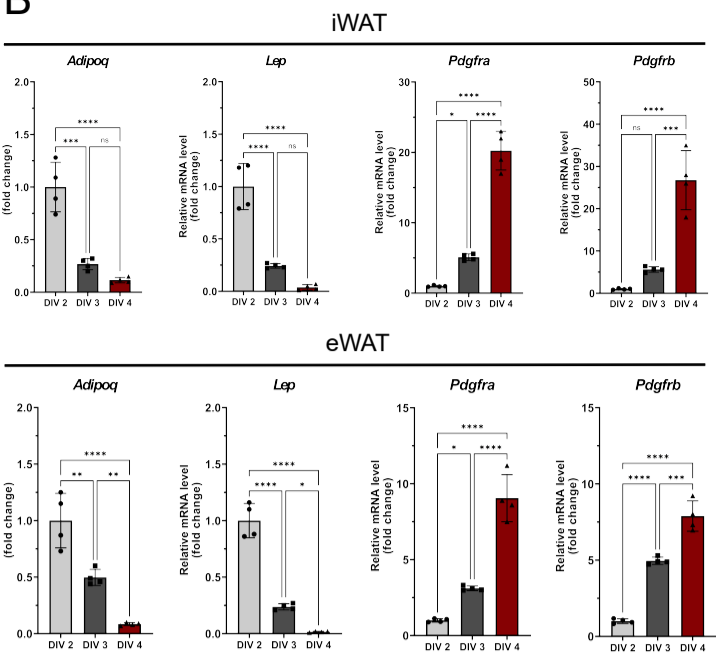

C

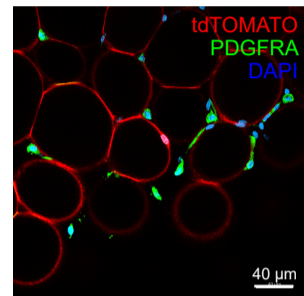

D

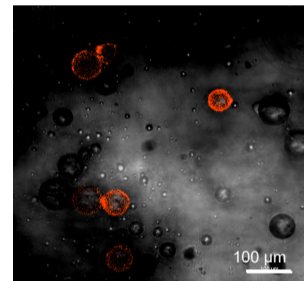

E

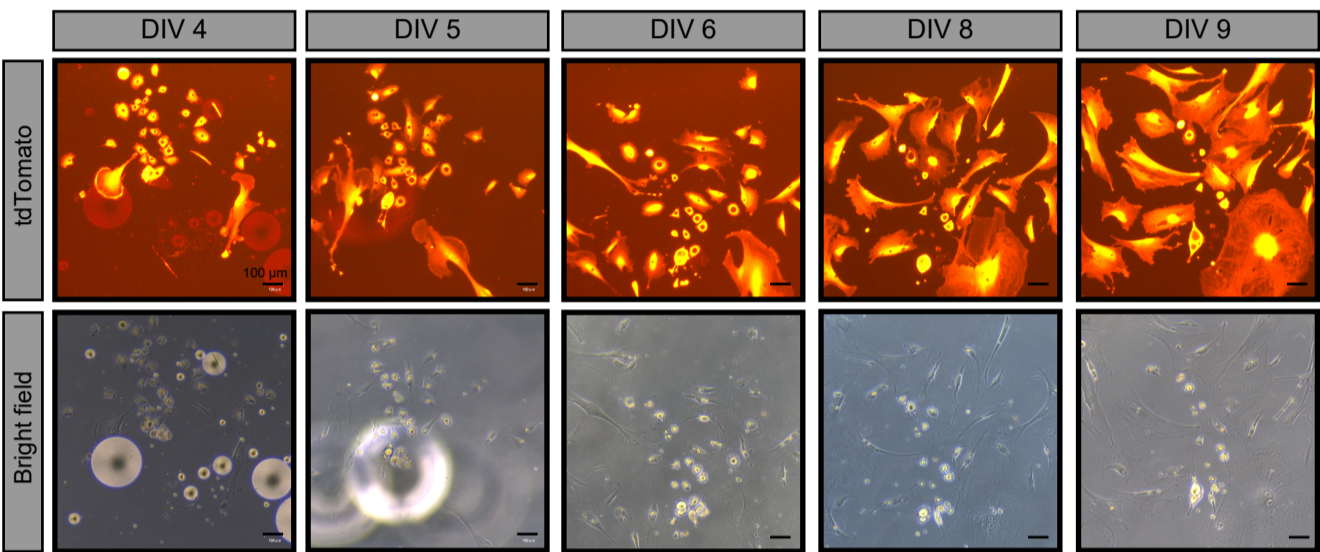

F

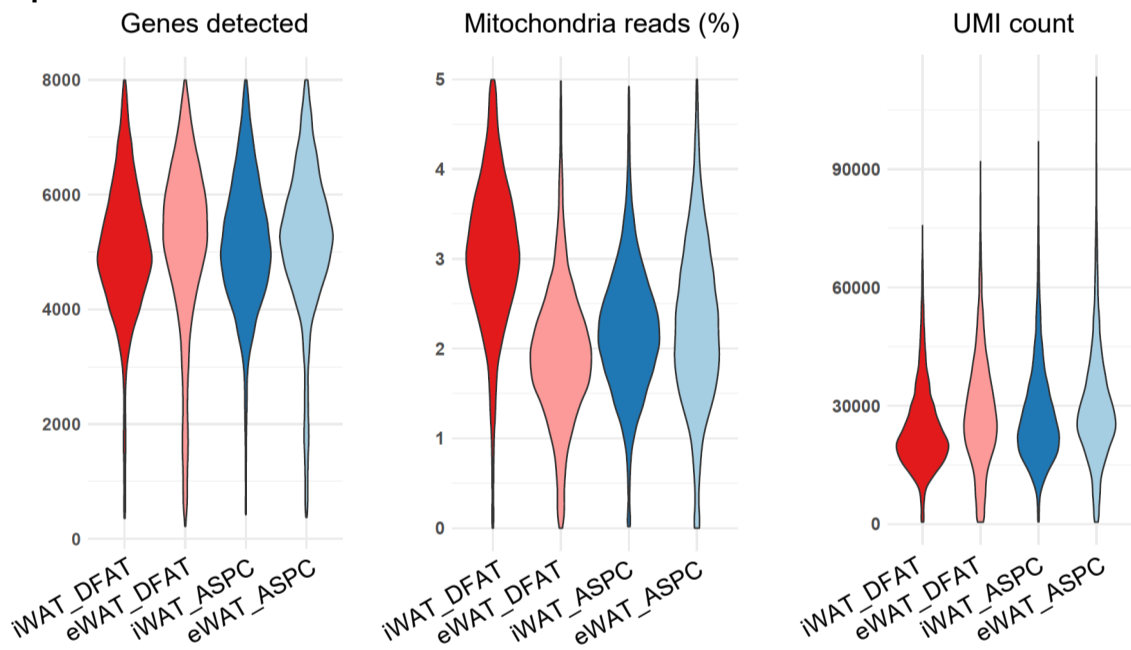

Supplement: Multimedia component 1 — Figure S1. Characterization of adipocyte de-differentiation in vitro. (A) Oil Red O staining of the adipocytes and DFAT cells along the de-differentiation process. Scale bar, 100 μm. (B) qPCR analysis of adipocyte (AdipoQ and Lep) and ASPC (Pdgfra and Pdgfrb) marker genes in iWAT (B) and eWAT (C) DFAT cells. Statistical significance was assessed by ordinary one-way ANOVA. ∗p < 0.05, ∗∗p < 0.01, ∗∗∗p < 0.001, ∗∗∗∗p < 0.0001. n = 4 independent flasks from 2 independent experiments, represented by a dot in the graph. Values were normalized to the DIV2 group and were presented as mean ± SD. (C) PDGFRA antibody staining (green) and tdTomato signal (red) in whole-mount eWAT tissue section of AdipoQCreERT2; Ai14 mice. Scale bar, 40 μm. (D) Presence of tdTomato + adipocytes in the upper layer of digested eWAT tissue of AdipoQCreERT2; Ai14 mice. Scale bar, 100 μm. (E) Daily monitoring of tdTomato + adipocytes during the in vitro de-differentiation process. The same visual field was followed in all images. DIV4 was one day after flipping the flask. Scale bar, 100 μm. (F) Quality controls of scRNA-seq of in vitro-derived DFAT cells and ASPCs. UMI, unique molecular identifier. [file mmc1.pdf]

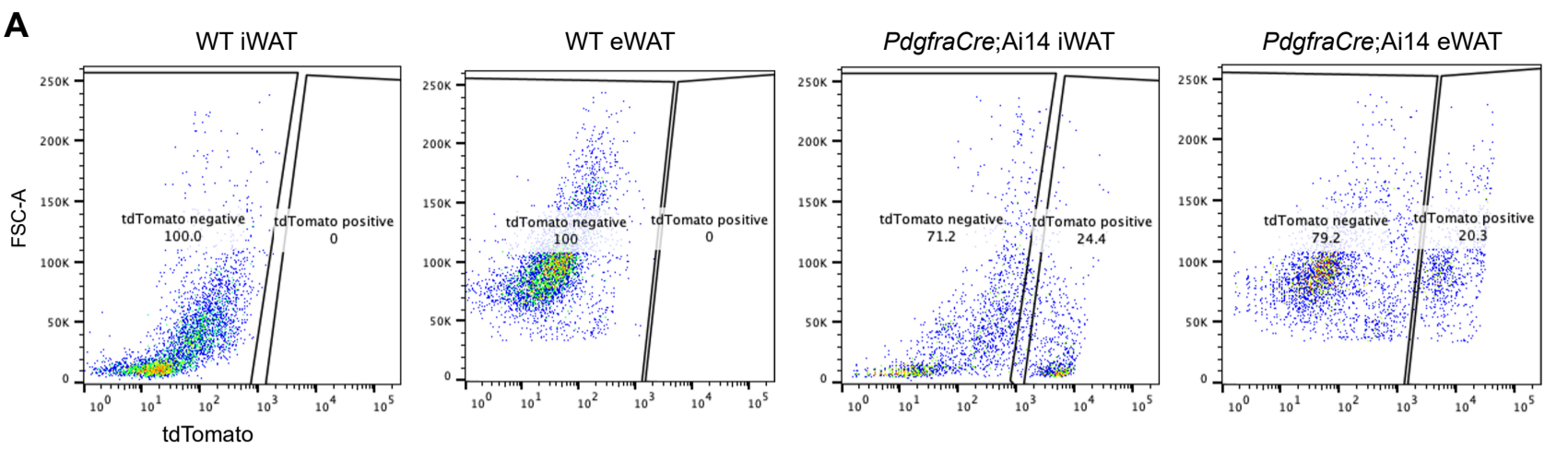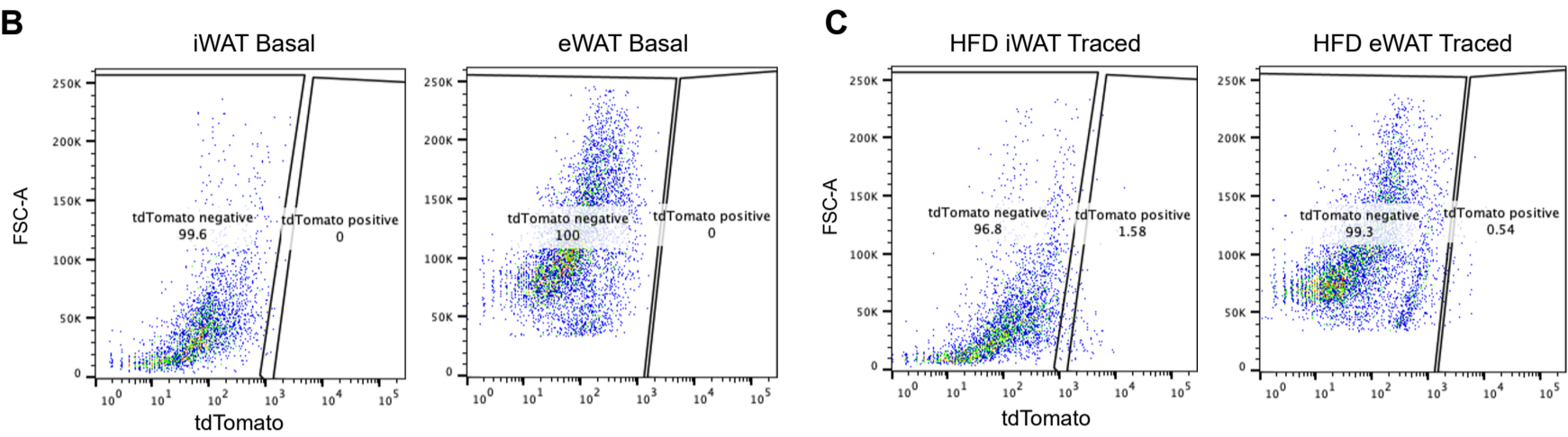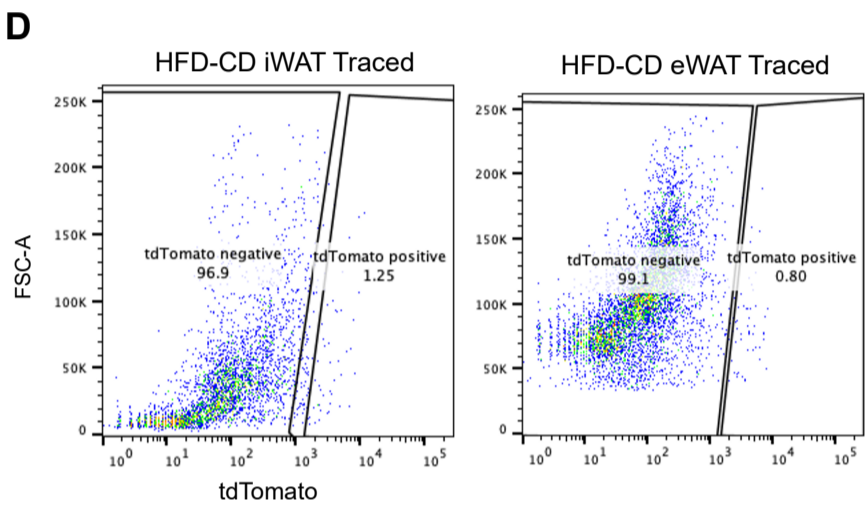

Supplement: Multimedia component 2 — Figure S2. FACS analysis of the traced DFAT cells (A) FACS gating setup with SVF cells from WT animals that do not express any tdTomato signal and PdgfraCre;Ai14 animals where all their ASPCs express tdTomato signal. (B–D) FACS analysis of tdTomato+ DFAT cells 4 days (B) or 6 weeks (C–D) after tamoxifen injection. Numbers in the diagrams indicate the percentage of the gated cell population in (A–D). [file mmc2.pdf]

**A**

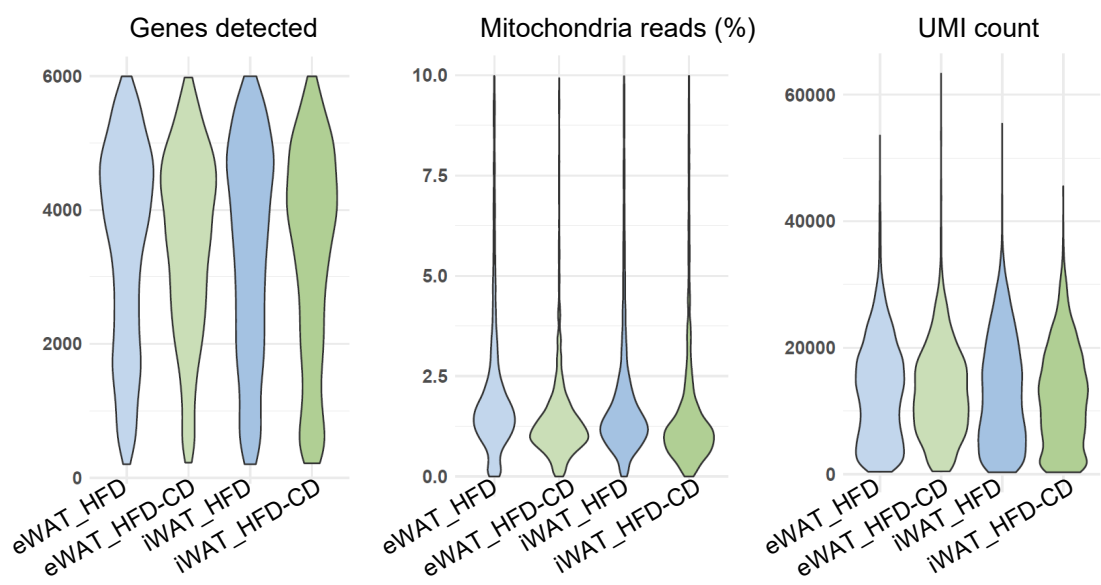

**B**

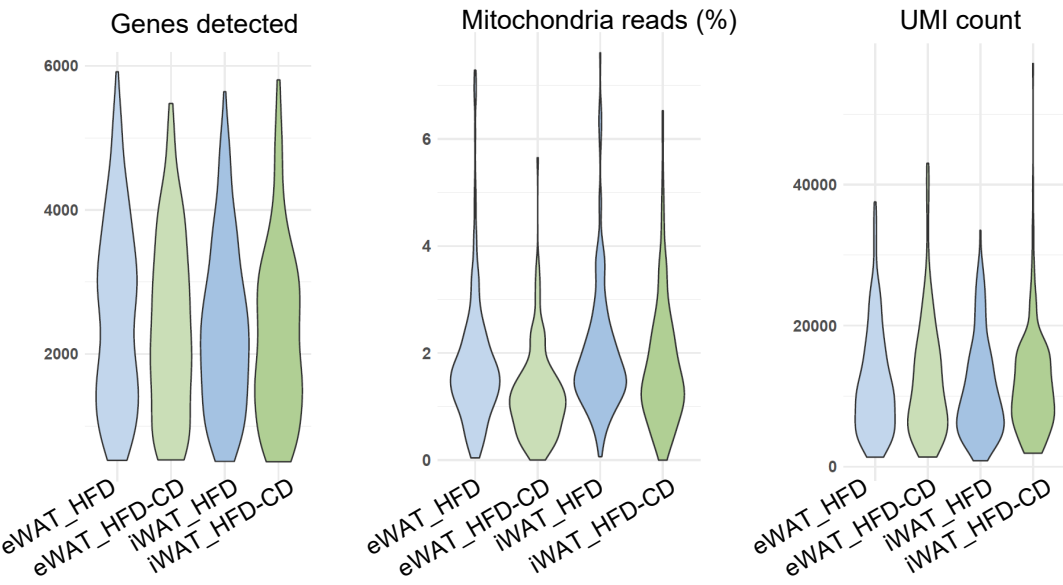

**C**

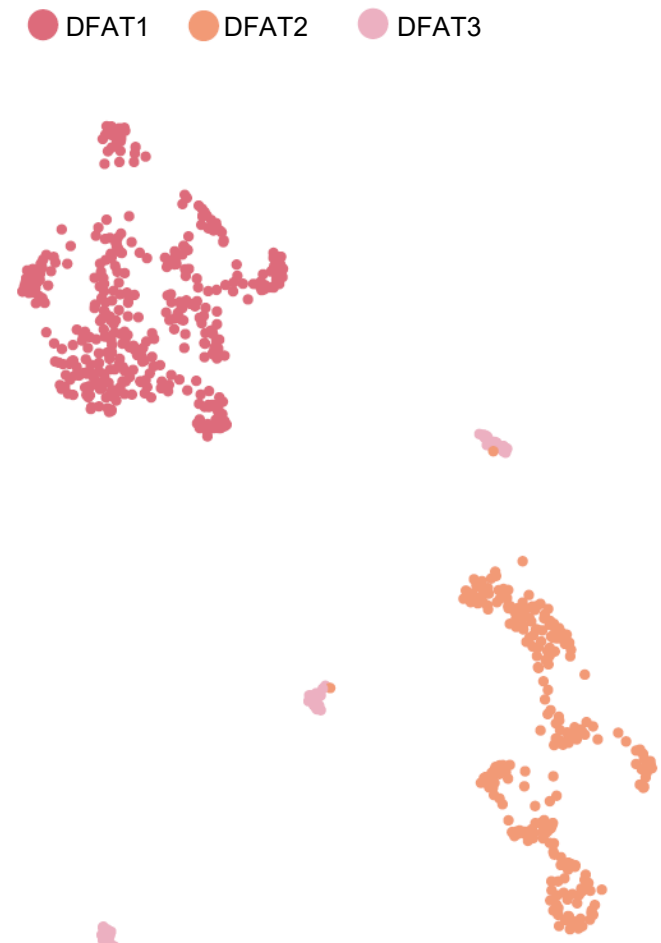

**D**

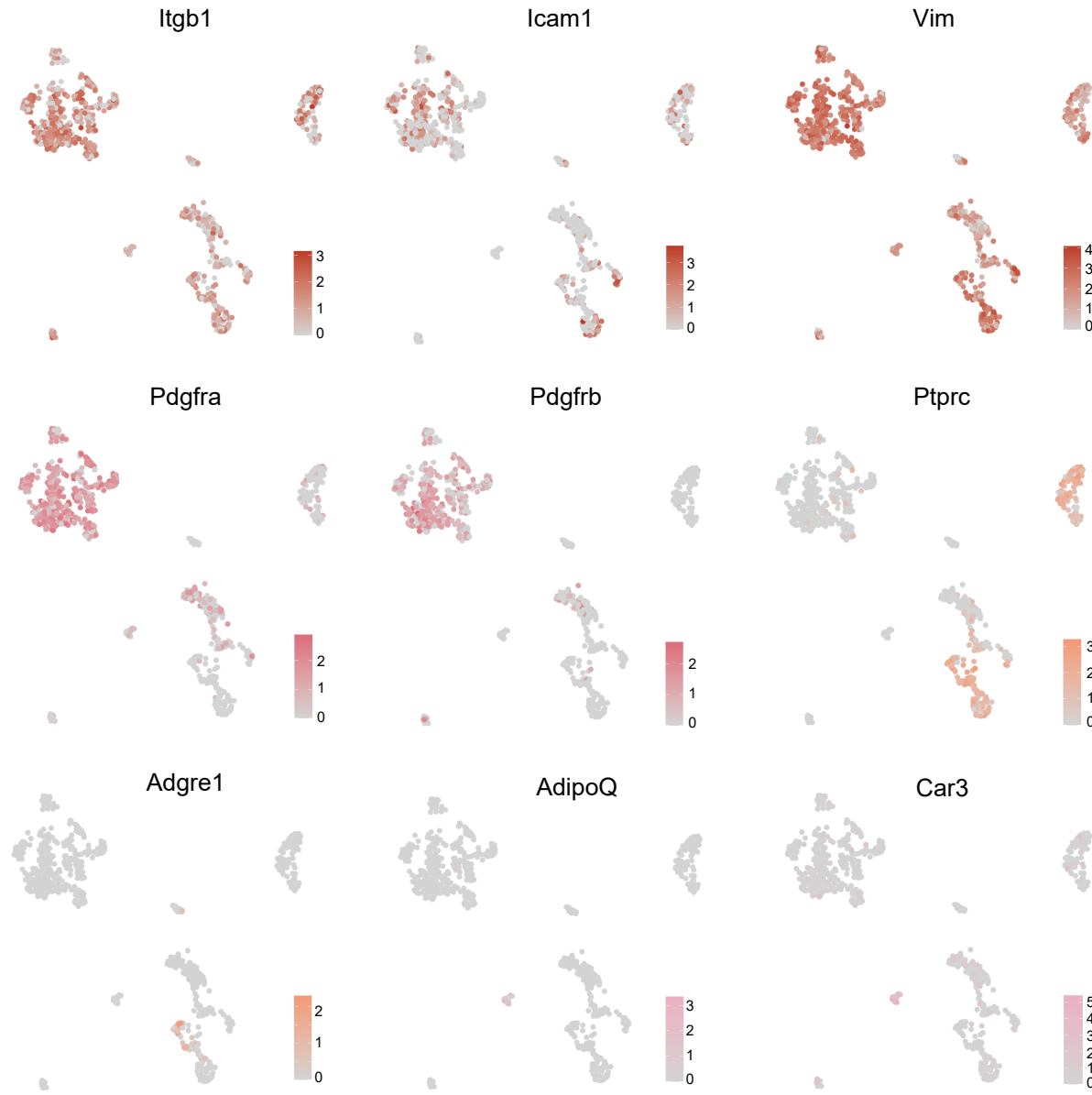

Supplement: Multimedia component 3 — Figure S3. Analysis of traced DFAT cells by Smart-seq3. (A–B) Quality controls of traced DFAT cells analyzed by scRNA-seq (A) and Smart-seq3 (B). (C) UMAP of the three DFAT cell sub-populations analyzed by Smart-seq3. (D) Feature plots showing the Smart-seq3-analyzed expression patterns of the same genes as in Figure 5F–I. [file mmc3.pdf]

**A**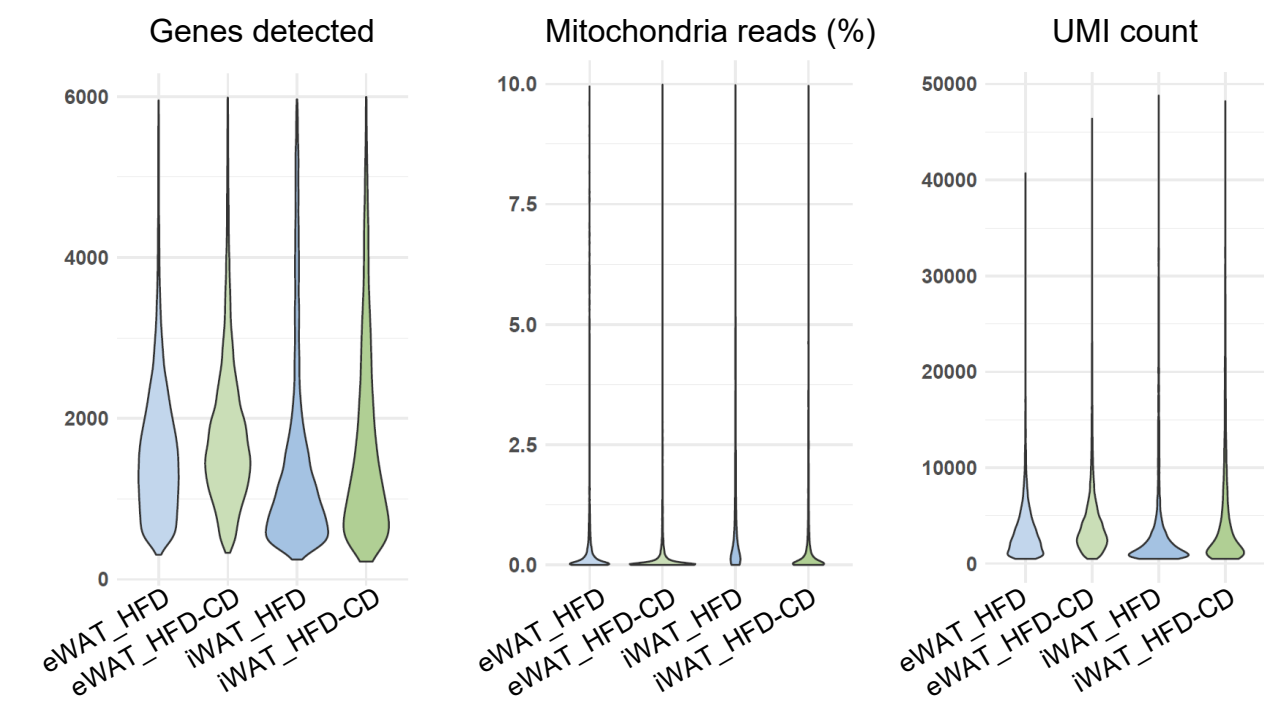**B**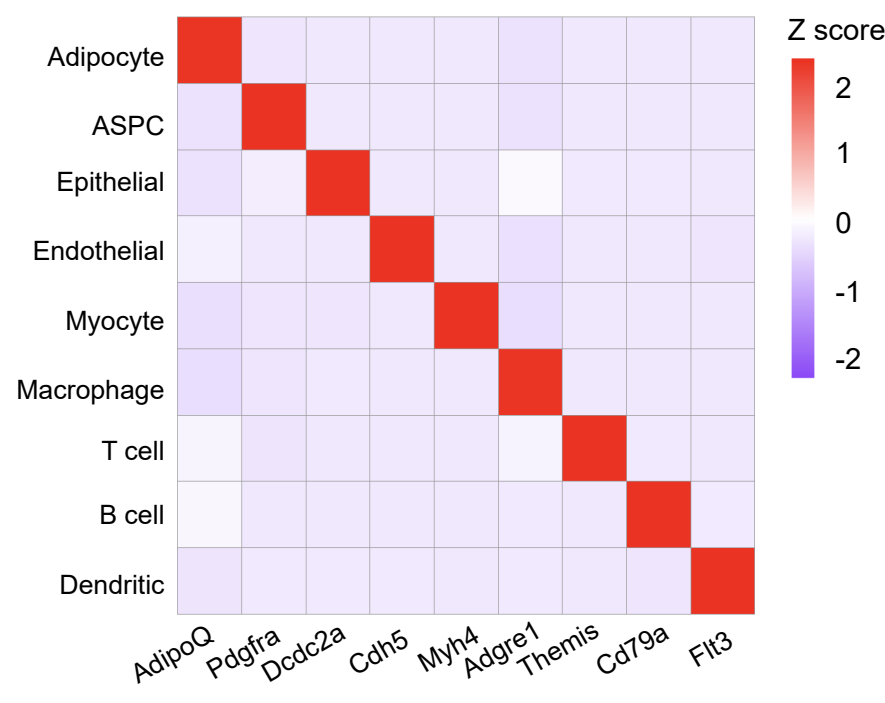**C**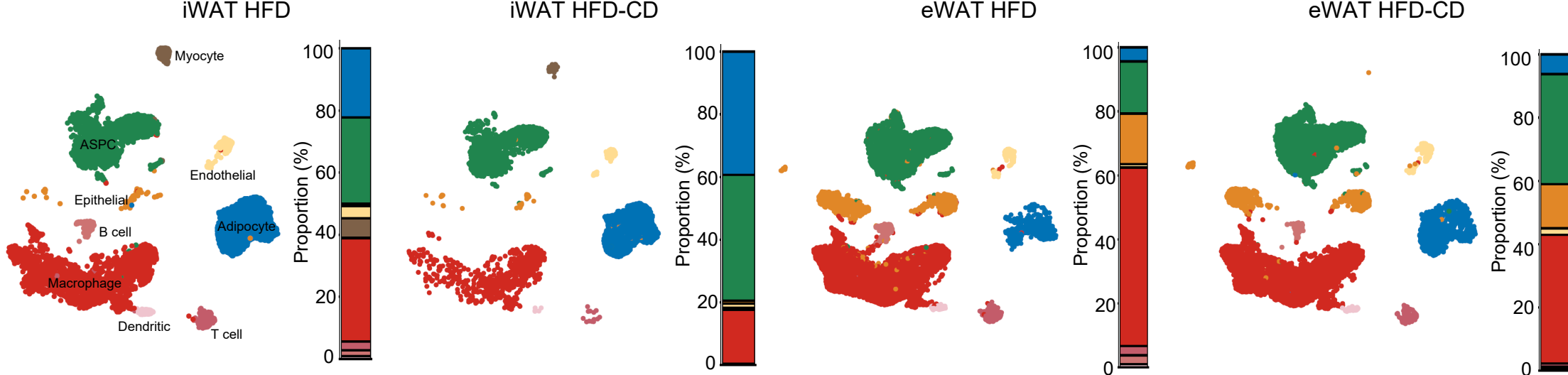**D**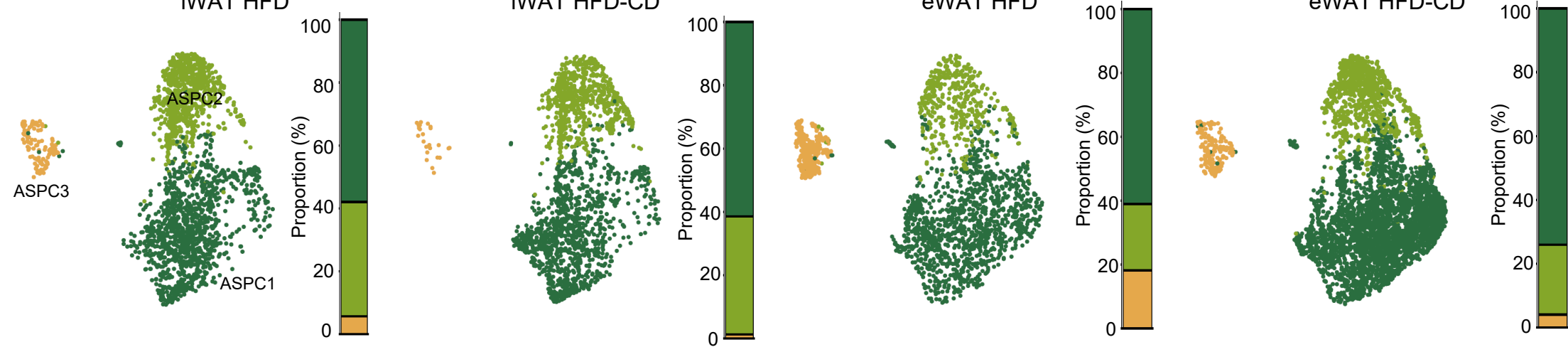**E**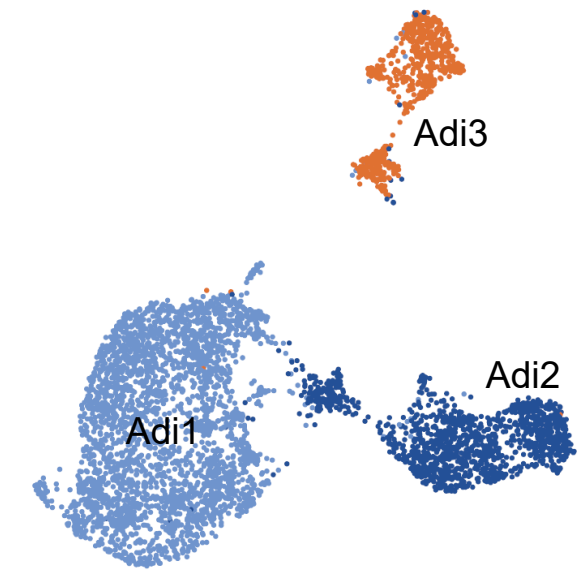**F**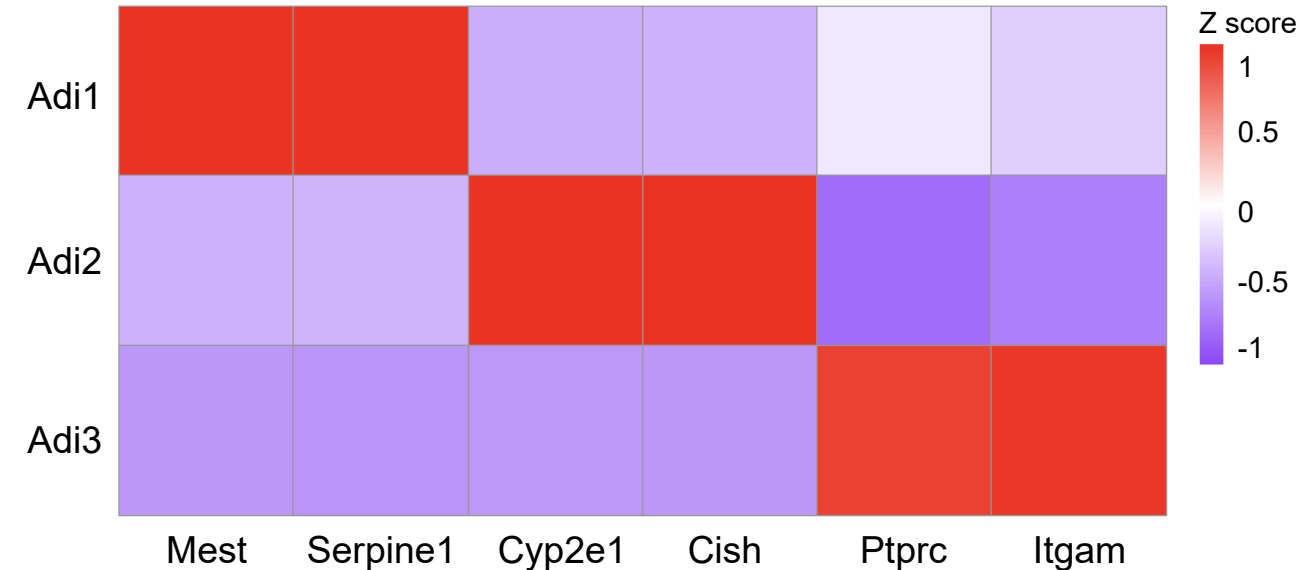**G**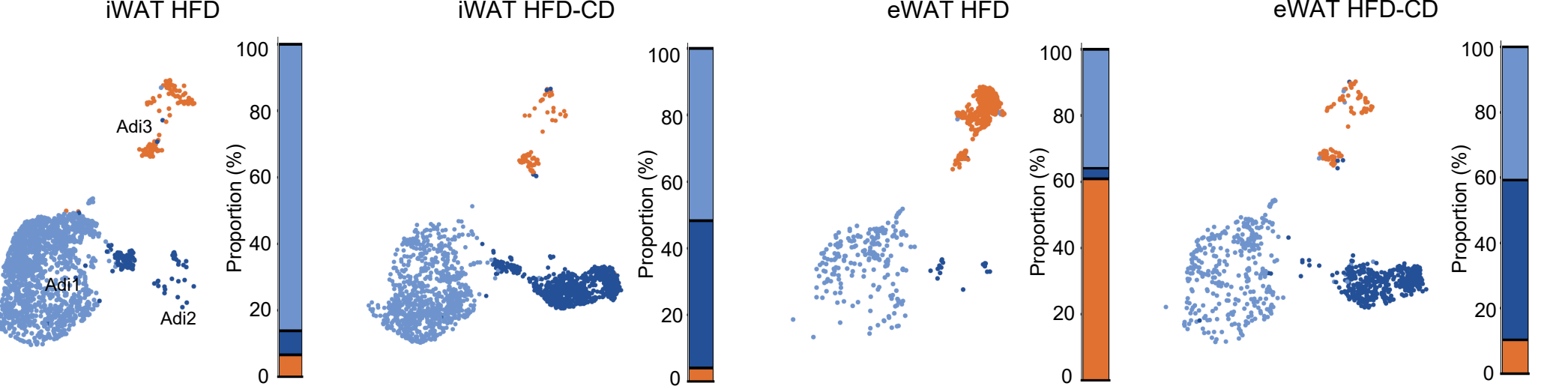**H**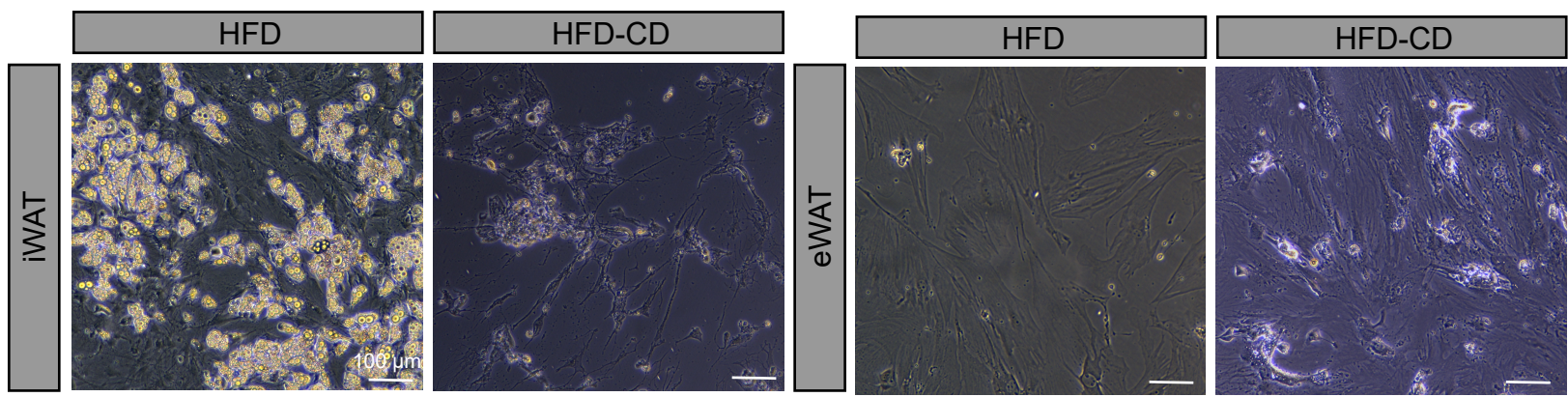

Supplement: Multimedia component 4 — Figure S4. Whole-tissue snRNA-seq analysis of iWAT and eWAT. (A) Quality controls of snRNA-seq of iWAT and eWAT from the two diet groups. (B) Heatmap showing the DEGs of each cell population in the integrated whole tissue snRNA-seq datasets from the two diet groups of iWAT and eWAT. (C) UMAPs and composition of each cell population in the four groups. (D) UMAPs and composition of each ASPC sub-population in the four groups. (E) UMAP of adipocyte sub-populations. (F) Heatmap showing the DEGs of each adipocyte sub-population. (G) UMAPs and composition of each adipocyte sub-population in the four groups. (H) Bright field images of DFAT cells sorted from iWAT and eWAT of the two diet conditions that have been cultured for adipocyte differentiation for 8 days. Scale bar, 100 μm. [file mmc4.pdf]
